# Refining the Amino Reactivity-Based Identification of Respiratory Sensitizers

**DOI:** 10.1021/acs.chemrestox.4c00545

**Published:** 2025-05-29

**Authors:** Martin Simoneit, Helene Langer, Nadin Ulrich, Alexander Böhme

**Affiliations:** UFZ Department of Exposure Science, 28342 Helmholtz Centre for Environmental Research, Permoserstraße 15, 04318 Leipzig, Germany

## Abstract

The sensitization of the respiratory tract may lead to various pulmonary diseases such as asthma. It can be triggered by the chemical reaction of organic electrophiles with nucleophiles of lung proteins with amino groups being of particular interest in this case. For assessing the dermal sensitization potential of chemicals, the direct peptide reactivity assay (DPRA) has become an OECD-accepted nonanimal test system. However, issues with the identification of known respiratory sensitizers such as isocyanates and anhydrides based on their amino reactivity in the DPRA have been reported. Hence, in this study the chemoassay employing glycine-*para*-nitroanilide (Gly-pNA) as model nucleophile is applied to eight iso­(thio)­cyanates, seven anhydrides, four dinitrobenzenes, one triazine, five acrylates, glutaraldehyde, and chloramine T to quantify their amino reactivity in terms of the second order rate constant *k*_Gly_ and the DPRA-like 24 h percent depletion *D*_Gly_. A comparison of *D*_Gly_ with respective DPRA amino reactivity data (*D*_DPRA_) showed that in particular iso­(thio)­cyanates and anhydrides are substantially more reactive toward Gly-pNA. This can be rationalized by the unintentional and so far not considered reaction of the test compounds with the ammonium acetate buffer used for DPRA testing. A detailed analysis of this reaction includes half-lives and analytically determined adduct patterns and indicates that it can hamper the envisaged depletion of the DPRA amino nucleophile. Finally, the obtained log *k*_Gly_ values range from −3.73 to ≥ 4.52 and allow for an improved identification of respiratory sensitizers. Hence, the Gly-pNA chemoassay may serve as a nonanimal screening method as one part of a mechanism-informed integrated testing and assessment strategy for respiratory sensitizers.

## Graphical abstract

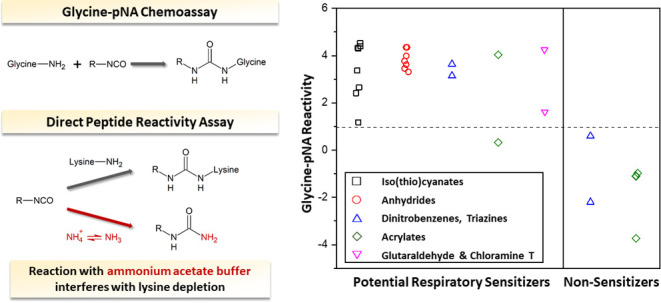

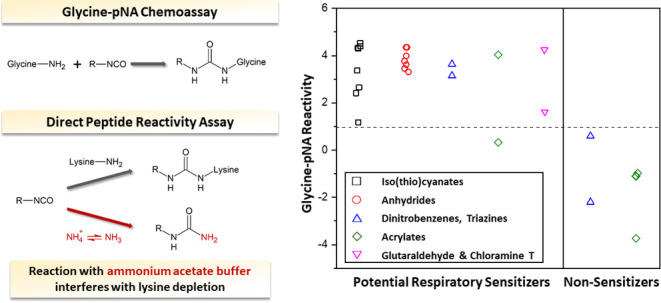

## Introduction

According to the European Chemical Agency (ECHA), the term respiratory sensitization describes various pulmonary diseases such as asthma, rhinitis, and extrinsic allergic alveolitis which are all caused by inhalation of chemicals.^1^ These so-called respiratory sensitizers cover large molecules (>5 kDa) such as wheat and rodent urinary proteins but also low-molecular weight chemicals (typically <0.5 kDa). The latter include organic electrophiles such as isocyanates, anhydrides, epoxides, and some acrylates.^2–5^ Moreover, many potential respiratory allergens have also been associated with plastic/polymer production and use,^6^ and inhalation of respective particles may also contribute to the development of respiratory sensitization.^7^

The mechanisms underlying respiratory sensitization are not fully elucidated yet. One route involves the priming of the immune system and is initiated by chemical reactions of low-molecular-weight organic electrophiles with lung proteins.^8,9^ These reactions may alter protein structure and trigger further key events including the identification of the protein-electrophile complex as allergens via recognition receptors, the expression of cell surface markers, chemokines, and type 2 cytokines (IL-4, IL-5, and IL-13), the activation and migration of dendritic cells, and an antibody-mediated immune response.^8–10^ Finally, Th2 immune cells proliferate in the lymph nodes and repeated exposure to an allergen leads to a hypersensitive immune response.

According to the European guideline on classification, labeling, and packaging of substances and mixtures, respiratory sensitizers need to be labeled with the signal word “danger”, the phrase “May cause allergy or asthma symptoms or breathing difficulties if inhaled”, and the GHS08 symbol.^11^ However, no accepted and validated methods specifically developed for the identification of respiratory sensitizers are available yet.^10,12–14^ This lack of suitable assessment strategies has led to the situation that methods initially developed for testing of dermal sensitizers are used for this purpose because both types of sensitization share mechanistic similarities.^4,8,10,12–15^ One example is the murine local lymph node assay (LLNA), the OECD gold standard animal test for the assessment of the skin sensitization potential of chemcials.^16^ The LLNA has been further refined to mimic the uptake of potential sensitizers through the respiratory tract (respiratory LLNA).^17^ However, it measures the overall T cell proliferation from the lymph nodes^16^ but does not distinguish between the two different types of T cell responses triggered by respiratory (Th-2 response) and dermal allergens (Th-1 response), respectively. Hence, the respiratory LLNA typically detects both, respiratory and dermal allergens.^12,17^

Driven by animal well-fare and high costs of animal tests, in chemico,^4,18–20^ in vitro,^13,21–23^ and in silico methods^2,3,24–28^ have been developed to assess the respiratory sensitization potential of chemicals guided by the respective adverse outcome pathway (AOP).^9^ These methods, however, are not entirely validated and accepted by regulators inter alia because they do not allow for a sufficient identification of respiratory sensitizers by discriminating them from dermal sensitizers.^12,13^ One prominent example is the direct peptide reactivity assay (DPRA) which uses two synthetic peptides containing either cysteine (SH group) or lysine (ε-NH_2_ group) to mimic the chemical reaction of a low-molecular-weight electrophile with SH and NH_2_ groups of proteins.^29–31^ In the case of respiratory sensitizers, it was expected that they would show a reactivity preference for lysine-NH_2_ ^2,3,18–20^ because cysteine-SH might be less abundant in the oxygen-rich lung. According to the principle of hard and soft acid and bases (HSAB),^32–34^ respiratory sensitizing anhydrides and isocyanates are hard electrophiles, preferring reactions with harder nucleophiles like lysine-NH_2_. However, it has been shown that the HSAB principle often fails in predicting the reactivity preferences of organic electrophiles toward specific nucleophiles.^35,36^ DPRA analyses for respiratory sensitizers confirmed doubt on the general applicability of the HSAB principle as they indicated that only anhydrides showed a high lysine-NH_2_ preference. At the same time, isocyanates are less reactive toward lysine-NH_2_ but highly reactive toward the soft cysteine-SH group, resulting in a higher reactivity preference for the latter.^4,18–20^ The low lysine-NH_2_ reactivity of isocyanates was explained by their hydrolytic degradation at the used pH level of 10.2.^4,20^ Anhydrides are also known to be sensitive to hydrolysis^37^ but showed a clear lysine-NH_2_ reactivity preference. Overall, it was demonstrated that the DPRA could not identify respiratory sensitizers based on their reactivity preference toward NH_2_ groups.^4,18–20^

Another factor contributing to the degradation of the electrophilic test compounds in the DPRA setup could be the ammonium acetate buffer (100 mM) used as the aqueous reaction medium.^29–31^ At a pH level of 10.2, ammonia (NH_3_) is more abundant than ammonium (NH_4_^+^) (90 mM: 10 mM, *pK*_a_ = 9.25),^38^ and NH_3_ as a nucleophile could react with the electrophilic test compounds. This has not been systematically analyzed yet but our previous study on aldehydes already indicated a higher amino reactivity in an ammonium acetate free setup (as compared to the classic DPRA).^39^

Hence, the main goal of this work was to analyze whether or not the DPRA ammonium acetate buffer can interfere with the assessment of the amino reactivity of electrophilic compounds, and thus may hamper the identification of respiratory sensitizers and their discrimination from dermal allergens. To this end, we employed our Gly-pNA chemoassay^39,40^ to profile the amino reactivity of eight isocyanates, seven anhydrides, five electrophiles classified as reactive via nucleophilic substitution (dinitrobenzenes and triazines), five acrylates, glutaraldehyde, and chloramine T in terms of the second order rate constant *k*_Gly_. The results show that in contrast to the DPRA literature data,^4,18–20^ isocyanates are typically most reactive, followed by the anhydrides. Moreover, a comparison of newly determined DPRA-type 24 h percent depletion for the reactions of the 27 test compounds with Gly-pNA (*D*_Gly_) and Nα-acetyl-lysine-pNA (Lys-pNA, *D*_Lys_), respectively, with DPRA literature data (*D*_DPRA_) further strengthens the hypothesis that the ammonium acetate buffer directly reacts with the test compounds, which was further proven by profiling the stability of all test compounds in the ammonium acetate buffer in terms of half-lives (*t*_1/2_) and the detection of the respective test compound ammonia adducts. Finally, the applicability of the Gly-pNA chemoassay as a nonanimal alternative for the identification of respiratory sensitizers as well as their discrimination from dermal allergens is demonstrated.

## Materials & Methods

The eight iso­(thio)­cyanates, seven anhydrides, four nitrobenzenes, one triazine, five acrylates, glutaraldehyde, and chloramine T used in this study were provided by Sigma-Aldrich (Munich, Germany), Merck (Darmstadt, Germany), Alfa Aesar (Karlsruhe, Germany), ABCR GmbH (Karlsruhe, Germany), or TCI (Eschborn, Germany). The purity of almost all test compounds was at least 97%; only for the isomeric mixture of methyltetrahydrophthalic anhydride purity was lower (>80%) and glutaraldehyde was provided as an aqueous solution (50%). The chemical structures of all test compounds are shown in Scheme 1.

**Scheme 1:**
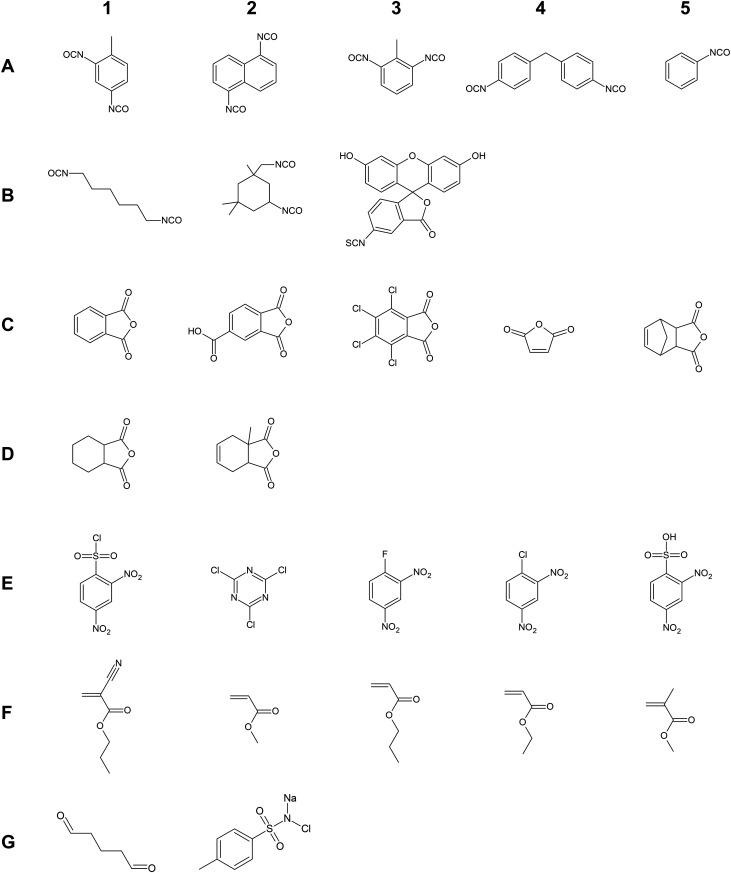
Chemical Structures of the 27 Test Compounds^a^

a (A1) 2,4-Toluene diisocyanate, (A2) 1,5-naphthalene diisocyanate, (A3) 2,6-toluene diisocyanate, (A4) 4,4′-methylene diphenyl diisocyanate, (A5) phenyl isocyanate, (B1) hexamethylene diisocyanate, (B2) isophorone diisocyanate, (B3) fluorescein isothiocyanate, (C1) phthalic anhydride, (C2) trimellitic anhydride, (C3) tetrachlorophthalic anhydride, (C4) maleic anhydride, (C5) himic anhydride, (D1) hexahydrophthalic anhydride, (D2) methyltetrahydrophthalic anhydride (isomeric mixture), (E1) 2,4-dinitrobenzenesulfonyl chloride, (E2) 2,4,6-trichloro-1,3,5-triazine, (E3) 1-fluoro-2,4-dinitrobenzene, (E4) 1-chloro-2,4-dinitrobenzene, (E5) 2,4-dinitrobenzenesulfonic acid, (F1) ethyl 2-cyanoacrylate, (F2) methyl acrylate, (F3) butyl acrylate, (F4) ethyl acrylate, (F5) methyl methacrylate, (G1) glutaraldehyde, and (G2) chloramine T.

Glycine-*para*-nitroanilide (Gly-pNA) and *N*-α-acetyl-lysine-*para*-nitroanilide (Lys-pNA), used as amino model nucleophiles were obtained from Bachem AG (Bubendorf, Switzerland). Potassium dihydrogen phosphate (anhydrous), sodium hydroxide solution (8 M, p.a. grade), ammonium acetate (LC grade), and formic acid (p.a. grade) were provided by Merck (Darmstadt, Germany). HPLC grade acetonitrile (ACN) was obtained from VWR International (Germany). Doubly distilled water was obtained from a “GFL 2104” distillation apparatus (GFLmbH, Germany).

### Selection and Classification of Test Compounds

The 27 test compounds used for this study have been mainly selected based on three reference lists for respiratory sensitizers^4,5,41^ and further literature classifying the test compounds as potential respiratory sensitizers or not.^42,43^ The aim was to set up a set of compounds covering potential respiratory sensitizers, nonsensitizers, and a diverse set of reaction types (acylation, nucleophilic substitution, Michael addition, Schiff base formation). In the Supporting Information Table S1 summarizes all criteria taken from the literature that have been used to classify the 27 compounds as either potential respiratory sensitizers or nonsensitizers. The group of potential sensitizers covers all isocyanates (A1–B3) and anhydrides (C1–D2), 2,4-dinitrobenzenesulfonyl chloride (E1), 2,4,6-trichloro-1,3,5-triazine (E2), ethyl 2-cyanoacrylate (F1), glutaraldehyde (G1), and chloramine T (G2). All other compounds were classified as nonsensitizers indicating that these compounds do not trigger immune-response-based respiratory diseases but does not exclude that these compounds can act as potential dermal sensitizers.

### Kinetic Gly-pNA Chemoassay

The reactivity of the 27 test compounds toward Gly-pNA was quantified in terms of the second-order reaction rate constant *k*_Gly_ using our Gly-pNA chemoassay.^39,40^ In brief, stock solutions of the test compound were freshly obtained every day by gravimetrically adding the required amount into a 10 mL volumetric flask, which was subsequently filled to volume with acetonitrile (ACN). The required amounts of test compounds depended on their reactivity toward Gly-pNA. Fast reacting test compounds were applied at lower concentrations than less reactive candidates to achieve a depletion of Gly-pNA that was detectable for our chromatographic analysis of the samples (not too fast) but time-efficient (not too slow). The final concentrations of the stock solutions ranged from 0.21 mM (for 1,5-naphthalene diisocyanate, A2) to 250 mM (for methyl methacrylate, F5). As usual for kinetic studies, fast reacting chemicals are applied at lower concentrations than little reactive candidates. All stock solutions were stored at 25 °C in a climate chamber before first use (ca. 30 min) and for repeating an experiment (max. 6 h). Gly-pNA stock solution (1.69 mM) was obtained by dissolving 3.3 mg Gly-pNA in 10 mL phosphate buffer solution (80 mM) and could be used for several weeks if stored at 4 °C. For all chemoassay experiments, 1.8 mL glass vials were used as reaction batches, which were initially filled with 1070 μL phosphate buffer (80 mM) and 30 μL Gly-pNA stock solution. To start the reaction, 400 μL of the test compound stock solutions was added. The depletion of Gly-pNA over the course of the reaction with the test compound was recorded using an HPLC 1200 infinity system from Agilent (Santa Clara, USA) as described in detail previously.^39,40^ A sequence for determining *k*_Gly_ typically contains two blank runs (no injection), three runs for the initial Gly-pNA concentration (no reaction with the test compound, used as controls), and three to ten analyses of the reaction mixture. The interval between two analyses of a reaction mixture ranged from 6 min (fast reacting compounds) up to 4 d (slowly reacting methyl methacrylate; F5). The time between the start of the reaction and the first analysis was measured with an electronic time clock. All reactions were performed under pseudo-1st order conditions with the electrophile being at least ten times in excess over Gly-pNA.

### Determination of *k*_Gly_

The isocyanates A1–A5, the anhydrides C1–C4, 2,4-dinitrobenzenesulfonyl chloride (E1), and 2,4,6-trichloro-1,3,5-triazine (E2) showed very high reactivity with Gly-pNA and the chromatographic signal of Gly-pNA already showed a substantial decrease after the first measurement (see Figure S1). For these compounds, the minimum reactivity was estimated in terms of *k*_Gly_ by plotting ln (*A*_Gly_^*t*^/*A*_Gly_^0^) vs reaction time *t* (with *A*_Gly_^*t*^ and *A*_Gly_^0^ being the peak areas under the chromatographic signal for Gly-pNA at time *t* and at the beginning of the reaction (*t* = 0)). Linear regression through 0 (at *t* = 0) and the first measurement point yields slope *s* (see Figure S1) which is the pseudo-1st order rate constant *k*_Gly_^pseudo^ that can be converted with the initial test compound concentration in the reactions mixture (*c*) into *k*_Gly_ according to eq 1.^39^

1 $$k_{Gly}=k_{Gly}^{pseudo}/c$$

For the reactions of Gly-pNA with the isocyanates B1 and B2, the anhydrides C5–D2, and methyl methacrylate (F5), respectively, curved trends could be observed when plotting ln (*A*_Gly_^*t*^/*A*_Gly_^0^) vs reaction time *t* (see Figure S2), indicating a loss of the test compound (e.g., through hydrolysis) over the course of the reaction time, which is not caused by the reaction with the Gly-pNA.^44,45^ To account for this loss, *k*_Gly_ was determined through our previously described polynomial approach given by eq 2 (see Figure S2).^45^

2 $$\mathrm{Ln}\left(A_{Gly}^{t}/A_{Gly}^{0}\right)\approx -k_{Gly}\cdot c\cdot t+0.5\cdot k_{Gly}\cdot k_{loss}\cdot c\cdot t^{2}$$

Here *k*_loss_ is the first order rate constant covering all processes contributing to the loss of the test compounds, except the reaction with Gly-pNA. The regression parameter of the linear part of eq 2 (= *k*_Gly_·*c*) is the pseudo-1st order rate constant *k*_Gly_^pseudo^ and can be converted into *k*_Gly_ according to eq 1 (see above).

For the remaining nine compounds (B3, E3–E5, F1–F4, G2) *k*_Gly_ was determined by plotting ln (*A*_Gly_^*t*^/*A*_Gly_^0^) vs reaction time *t* (see Figure S3). Linear regression of these plots provides slope *s*, which is again the pseudo-1st order rate constant, *k*_Gly_^pseudo^, and can be used to calculate *k*_Gly_ according to eq 1. All experiments to determine *k*_Gly_ have been conducted at least three times. For glutaraldehyde (G1), *k*_Gly_ was taken from our former publication.^39^

### DPRA-Like Chemoassay with Gly-pNA & Lys-pNA

In addition to the kinetic Gly-pNA chemoassay, the reactivity of the 27 compounds toward Gly-pNA and Lys-pNA was determined in terms of percentage depletions after reacting for 24 h at 25 °C employing the following DPRA-like setup: stock solutions for the test compounds were prepared by gravimetrically adding at least 3 mg of the respective compound into a glass vial (total volume 1.8 mL) and subsequent addition of 1 mL ACN. The yielded stock solutions were further diluted in ACN to obtain a final test compound concentration of 5.77 mM in 1 mL ACN. The stock solutions for Gly-pNA and Lys-pNA with a final concentration of 1.7 mM were prepared by dissolving the required amount in one of the three aqueous buffer solutions used for this DPRA-like chemoassay: phosphate buffer (80 mM, pH 7.4), phosphate buffer (80 mM, pH 10.2), and ammonium acetate buffer (100 mM, pH 10.2). Glass vials (1.8 mL) were used as reaction vessels. More detailed information on the setups used are summarized in Table S2.

In the final reaction mixtures, the concentration of Gly-pNA or Lys-pNA was 0.03 mM, and the test compound was 50 times in excess (1.5 mM). After incubation for 24 h at 25 °C, the free amount of either Gly-pNA or Lys-pNA was determined using the HPLC UV–vis method described for the kinetic Gly-pNA chemoassay.^39,40^ The percentage depletion of Gly-pNA (*D*_Gly_) or Lys-pNA (*D*_Lys_) was determined directly from peak areas according to eq 3

3 $$D_{x}\;\left[\%\right]=\left(\frac{A_{x}^{control}-A_{x}}{A_{x}^{control}}\times 100\right)\;with\;x=Gly\;\text{or}\;Lys$$

In eq 3, *A*_*x*_ and *A*_*x*_^control^ are the peak areas of the chromatographic signal of either Gly-pNA (*x* = Gly) or Lys-pNA (*x* = Lys) at 315 nm after 24 h incubation with and without test compound, respectively.

### Stability Assessment of Test Compounds

The stability of the test compounds in the reaction media comprising (besides ACN) either aqueous phosphate buffer (80 mM, pH 7.4 or pH 10.2) or aqueous ammonium acetate buffer (100 mM, pH 10.2) were characterized in terms of respective half-lives (*t*_1/2_). To this end, the test compound was dissolved in 1 mL ACN in a glass vial. The required amount of test compound was typically between 1 and 10 mg, and mainly depended on the UV–vis detectability of the test compound. As reaction batches, 1.8 mL glass vials equipped with 400 μL glass inserts were used and filled with 220 μL of the respective aqueous buffer and 80 μL of the test compound stock solution. The depletion of the test compounds was recorded with the HPLC UV–vis instrument used for the kinetic Gly-pNA chemoassay.^39,40^ Chromatographic separation of the reaction mixture was achieved using the gradient described for the Gly-pNA chemoassay previously^39,40^ and an isocratic HPLC method (water/ACN 70:30, both with 0.1 vol % formic acid). The wavelengths used for the detection of the compound and the information on the used chromatographic method are given in the Supporting Information in Table S3.

### Adduct Pattern Analysis Using HPLC-Tandem MS

Adducts formed by the reaction of the test compounds with ammonium acetate were analyzed directly from the reaction mixtures using a 1290 series HPLC system coupled to a G6460 QqQ mass spectrometer (both from Agilent, Santa Clara, USA) as positively or negatively charged ions (electron spray ionization) as described before.^39,40,46^ To avoid artificial formation of ammonium adducts in the ionization source, ammonium-free water and acetonitrile (both containing 0.1 vol % formic acid) were used as eluents.

## Results & Discussions

### Amino Reactivity of Test Compounds in Terms of *k*_Gly_

The reactivity of eight iso­(thio)­cyanates, seven anhydrides, four dinitrobenzenes, one triazine, five acrylates, glutaraldehyde, and chloramine T toward the amino group of Gly-pNA has been characterized in terms of the second order rate constant *k*_Gly_. Respective *k*_Gly_ data is summarized in Table 1 and range over more than eight orders of magnitude, from highly reactive 2,4-toluene diisocyanate (A1; log *k*_Gly_ ≥ 4.52) to less reactive methyl methacrylate (F5; log *k*_Gly_ = −3.73).

**Table 1:** Chemoassay Data for the Reaction of Glycine-*para*-Nitroanilide (Gly-pNA) with the 27 Test Compounds in Terms of the Second-Order Rate Constant *k*_Gly_

compound	no	*k*_Gly_ ± s(*k*_Gly_) [L mol^–1^ min^–1^]	log *k*_Gly_
Isocyanates & Isothiocyanates			
2,4-toluene diisocyanate^a^	A1	≥33484 ± 803	≥4.52
1,5-naphthaline diisocyanate^a^	A2	≥24928 ± 12821	≥4.40
2,6-toluene diisocyanate^a^	A3	≥20880 ± 695	≥4.32
4,4′-methylene diphenyl diisocyanate^a^	A4	≥20015 ± 4486	>4.30
phenyl isocyanate^a^	A5	≥2368 ± 309	≥3.37
hexamethylene diisocyanate^b^	B1	451 ± 33	2.65
isophorone diisocyanate^b^	B2	256 ± 6	2.41
fluorescein isothiocyanate	B3	15.1 ± 2.6	1.18
Anhydrides			
phthalic anhydride^a^	C1	≥22480 ± 1197	4.35
trimellitic anhydride^a^	C2	≥22271 ± 1975	4.35
tetrachlorophthalic anhydride^a^	C3	≥9816 ± 858	3.99
maleic anhydride^a^	C4	≥5930 ± 160	3.77
himic anhydride^b^	C5	4126 ± 114	3.62
hexahydrophthalic anhydride^b^	D1	2900 ± 110	3.46
methyltetrahydrophthalic anhydride	D2	2051 ± 32	3.31
S_N_Ar electrophiles (trianzines and dinitrobenzenes)			
2,4-dinitrobenzenesulfonyl chloride^a^	E1	≥4387 ± 22	≥3.64
2,4,6-trichloro-1,3,5-triazine^a^	E2	≥1424 ± 364	≥3.15
1-fluoro-2,4-dinitrobenzene	E3	3.95 ± 0.07	0.60
1-chloro-2,4-dinitrobenzene	E4	0.00638 ± 0.00023	–2.20
2,4-dinitrobenzenesulfonic acid	E5	not reactive	
Acrylates			
ethyl 2-cyanoacrylate	F1	2.22 ± 0.33	0.35
		10886 ± 1589^c^	4.04
methyl acrylate	F2	0.108 ± 0.005	–0.97
butyl acrylate	F3	0.0826 ± 0.0141	–1.08
ethyl acrylate	F4	0.0795 ± 0.0001	–1.10
methyl methacrylate^b^	F5	0.000185 ± 0.000019	–3.73
Others			
glutaraldehyde^d^	G1	17731 ± 2525	4.25
chloramine T	G2	41.9 ± 0.2	1.62

a Test compound for which *k*_Gly_ represents the minimum Gly-pNA (see Figure S1).

b *k*_Gly_ determined using polynomial regressions based on eq M2 (see Figure S2).

c *k*_Gly_ determined using the free (not bound by water) fraction of F1.

d *k*_Gly_ of G1 was taken from our previous work.^39^

Scheme 2 proposes the underlying reaction mechanisms.

**Scheme 2:**
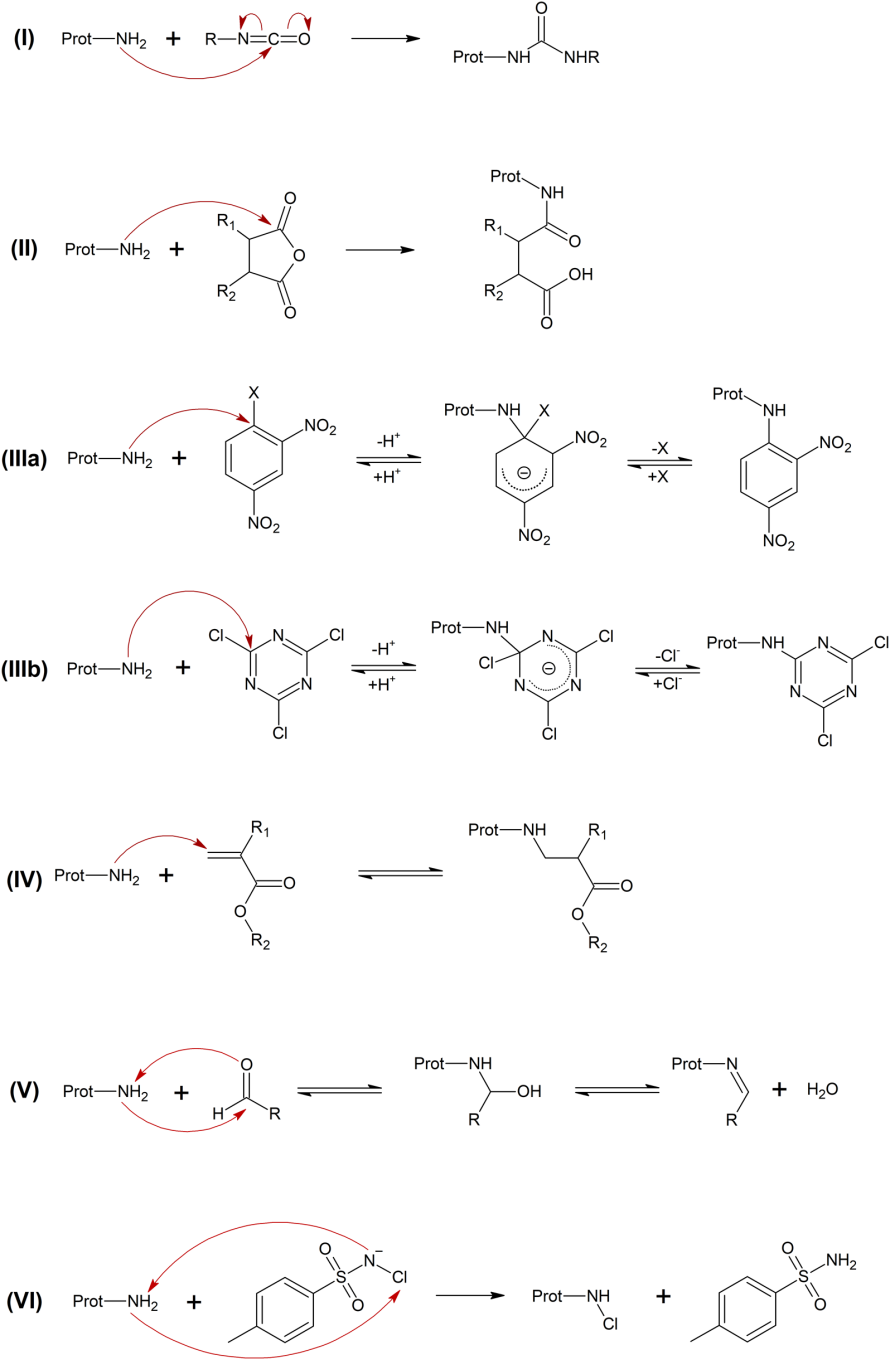
Mechanisms Underlying the Reaction of Amino Groups with Isocyanates (I), Anhydrides (II), Dinitrobenzenes (IIIa), Trichlorotriazines (IIIb), Acrylates (IV), Aldehydes (V), and Chloramine T (VI)

Within the subset of iso­(thio)­cyanates ((I) in Scheme 2), the aromatic isocyanates A1–A5 show highest Gly-pNA reactivity (log *k*_Gly_ ≥ 3.37), followed by the aliphatic isocyanates B1 and B2 (log *k*_Gly_: 2.65 and 2.41), and fluorescein isothiocyanate (B3, log *k*_Gly_ = 1.18). Note, that *k*_Gly_ for the aromatic isocyanates represents the minimum Gly-pNA reactivity of these test compounds because it was determined based on the initial Gly-pNA concentration and the first measurement after the start of the reaction (see above and Figure S1). Furthermore, isocyanates are sensitive toward hydrolysis (which will be later discussed in detail). This hydrolytic degradation is not addressed for the determination of *k*_Gly_ of A1–A5 due to the limited number of measurement data (see Figure S1). It leads to an overestimation of the reactive fraction of the aromatic isocyanates (A1–A5), and their true Gly-pNA reactivity is higher than given by the *k*_Gly_ values in Table 1. Finally, the comparatively low reactivity of fluorescein isothiocyanate (log *k*_Gly_ = 1.18) is caused by the lower electronegativity of sulfur which makes the isothiocyanate group less Gly-pNA reactive as compared to isocyanates.

Among the subset of anhydrides ((II) in Scheme 2), the aromatic candidates (C1–C4 with log *k*_Gly_ ≥ 3.77) are more reactive than the aliphatic anhydrides (C5–D2 with log *k*_Gly_ ≤ 3.62). This can be explained by the positive inductive effect of the aliphatic groups, which reduces the Gly-pNA reactivity of the carbonyl carbon.

Within the subset of S_N_Ar electrophiles ((IIIa/b) in Scheme 2), 2,4-dinitrobenzenesulfonyl chloride (E1) shows the highest Gly-pNA reactivity (log *k*_Gly_ ≥ 3.64). This high reactivity is caused by the attack of Gly-pNA at the sulfonyl chloride group (see Scheme S1).^47,48^ The trichlorotriazine E2 also shows high Gly-pNA reactivity (log *k*_Gly_ ≥ 3.15) due to the activation of the triazine ring by the three chlorine substituents for being attacked by nucleophilies.^49^

Within the group of acrylates ((IV) in Scheme 2), ethyl 2-cyanoacrylate (F1) is the most reactive (log *k*_Gly_ = 0.33). This higher reactivity is caused by the cyano group (C≡N), as clearly shown through the comparison of F1 with its cyano-free counterpart, ethyl acrylate (F4, log *k*_Gly_ = −1.1). However, it should be mentioned that cyanoacrylates can react with water (see Scheme S2, top).^50^ In our Gly-pNA chemoassay ethyl 2-cyanoacrylate was used in highly diluted solutions (< 0.0008 mol/L vs ≈ 40 mol/L water and ≈ 6.7 mol/L ACN), and thus predominantly occurs as water adduct that probably does not react with Gly-pNA. Hence, the *k*_Gly_^pseudo^ values used to calculate *k*_Gly_ of F1 (according to eq 1) only reflects the reaction of Gly-pNA with the minor amount of free (not bound to water) ethyl 2-cyanoacrylate and employing the nominal concentration of F1 to eq 1 overestimates the free (reactive) fraction leading to an underestimation of *k*_Gly_. In our chemoassay setup this free (reactive) fraction of F1 is <0.1% (further details are given in the Supporting Information), and considering this in eq 1 reveals a substantially higher *k*_Gly_ (10886 L·mol^–1^·min^–1^ ± 1589 L·mol^–1^·min^–1^). This shows that the amino reactivity of F1 can vary over orders of magnitude (in terms of log *k*_Gly_ from 0.35 to 4.04) depending on the concentration of water in the surrounding medium. Finally, glutaraldehyde ((V) in Scheme 2) showed high Gly-pNA reactivity (log *k*_Gly_ = 4.25) while chloramine T ((VI) in Scheme 2) was comparatively less reactive (log *k*_Gly_ = 1.62).

### Amino Reactivity of the Test Compounds in DPRA-Like Setups

The amino reactivity of the 27 test compounds has been further quantified in terms of 24 h percentage depletion for their reaction with Gly-pNA (*D*_Gly_) and Lys-pNA (*D*_Lys_), respectively, using a DPRA-like setup (see above and Table S2). To ensure that the amino groups of Gly-pNA (p*K*_a_ = 7.4)^51^ and Lys-pNA (p*K*_a_ = 10.4)^51^ are comparably protonated (= similar nucleophilic reactivity), pH 7.4 (phosphate buffer) was used for Gly-pNA while all experiments with Lys-pNA have been conducted at pH 10.2 (phosphate or ammonium acetate buffer). Here, it has to be mentioned that the buffer capacity of the phosphate buffer at pH 10.2 is comparatively low, and for some reactions pH level dropped substantially below 10 (see Supporting Information, Table S5). However, because phosphate buffer at pH 10.2 has been previously used in DPRA-type assays to assess the amino reactivity of potential sensitizers^52–54^ and to compare it with the ammonium acetate buffer, we used the phosphate buffer at pH 10.2 also for this study. Finally, data for the reaction of the test compounds with the DPRA lysine peptide (*D*_DPRA_, pH 10.2, ammonium acetate buffer) have been taken from the literature (if available).^4,30,55–57^ All depletion data is summarized in Table 2.

**Table 2:** DPRA-like Chemoassay Data for the Reaction of Glycine-*para*-Nitroanilide (Gly-pNA) and *N*-α-Acetyl Lysine-*para*-Nitroanilide (Lys-pNA), with the 27 Test Compounds in Terms of 24 h Percent Depletions (*D*_Gly_ and *D*_Lys_)­^a^ ^,^ ^b^

compounds	No	*D*_Gly_ ± *s*(*D*_Gly_) [ %]	*D*_Lys_ ± *s*(*D*_Lys_) [ %]		*D*_DPRA_ ± *s*(*D*_DPRA_) [ %]
		PPB pH 7.4	PPB pH 10.2	AAB pH 10.2	AAB pH 10.2
Isocyanates & Isothiocyanates					
2,4-toluene diisocyanate	A1	100	100	18.8 ± 0.6	22.0 ± 1.4
1,5-naphthaline diisocyanate	A2	100	100	19.9 ± 3.7	n.a
2,6-toluene diisocyanate	A3	100	100	20.7 ± 1.4	26.5 ± 0.7
4,4′-methylene diphenyl diisocyanate	A4	100	100	22.9 ± 4.4	23.0 ± 6.2
phenyl isocyanate	A5	100	100	10.2 ± 0.7	48.4 ± 1.1
hexamethylene diisocyanate	B1	100	98.7 ± 2.2	23.3 ± 0.3	41.6 ± 7.3
isophorone diisocyanate	B2	100	100	21.5 ± 0.3	28.0
fluorescein isothiocyanate	B3	100	100	56.5 ± 0.3	61.1
Anhydrides					
phthalic anhydride	C1	100	100	22.6 ± 7.4	69.2 ± 22.0
trimellitic anhydride	C2	100	100	15.2 ± 0.6	59.9 ± 22.0
tetrachlorophthalic anhydride	C3	100	99.2 ± 0.7	11.5 ± 1.8	83.5 ± 0.7
maleic anhydride	C4	100	100	12.0 ± 1.1	54.0 ± 25.5
himic anhydride	C5	100	98.9 ± 0.1	29.6 ± 0.3	n.a
hexahydrophthalic anhydride	D1	100	100	12.5 ± 0.5	56.4 ± 29.4
methyltetrahydrophthalic anhydride	D2	100	100	18.0 ± 0.9	n.a
S_N_Ar electrophiles (trianzines and dinitrobenzenes)					
2,4-dinitrobenzenesulfonyl chloride	E1	56.1 ± 0.5	62.6 ± 4.2	13.4 ± 0.8	n.a
2,4,6-trichloro-1,3,5-triazine	E2	100	100	91.1 ± 1.1	99.7 ± 0.6
1-fluoro-2,4-dinitrobenzene	E3	72.7 ± 0.7	100	84.0 ± 3.3	n.a
1-chloro-2,4-dinitrobenzene	E4	3.5 ± 0.6	8.1 ± 0.5	5.0 ± 0.7	21.1 ± 16.3
2,4-dinitrobenzenesulfonic acid	E5	5.9 ± 0.3	0	0.9 ± 0.1	27.4
Acrylates					
ethyl 2-cyanoacrylate	F1	98.3 ± 0.1	40.2 ± 1.9	1.6 ± 0.2	n.a
methyl acrylate	F2	16.6 ± 0.4	67.9 ± 1.6	55.5 ± 0.7	86.5 ± 5.2
butyl acrylate	F3	15.2 ± 0.2	74.4 ± 1.5	61.3 ± 0.4	87.8 ± 3.8
ethyl acrylate	F4	14.4 ± 0.6	67.1 ± 1.4	55.8 ± 0.4	90.3 ± 6.6
methyl methacrylate	F5	4.7 ± 0.7	2.6 ± 1.3	0.7 ± 0.3	10.6 ± 0.8
Others					
glutataraldehyde	G1	100^c^	97.9 ± 0.6	7.2 ± 1.7	82.8 ± 7.6
chloramine T	G2	100	53.8 ± 2.9	23.9 ± 1.6	80.8 ± 30.7

a As reaction media, either phosphate buffer (PPB; pH 7.4 or 10.2) or ammonium acetate buffer (AAB; pH 10.2) were used. Moreover, literature data for the depletion of the DPRA lysine peptide (*D*_DPRA_) through the reaction with the test compounds (*D*_DPRA_) are given (if available).

b *D*_DPRA_ data taken from literature.^4,30,55–57^ .

c *D*_Gly_ data taken from our previous work.^39^

As can be seen from Table 2, the isocyanates A1–B3, the anhydrides C1–D2, 2,4,6-trichloro-1,3,5-triazine (E2), glutaraldehyde (G1), and chloramine T (G2) fully depleted Gly-pNA within 24 h (*D*_Gly_ = 100%). Ethyl 2-cyanoacrylate (F1) also depletes Gly-pNA almost entirely (*D*_Gly_ = 98.3%). Moderate *D*_Gly_ values are observed for the dinitrobenzenes E1 and E3 (56.1% and 72.7%) although for E1 a large *k*_Gly_ value (4126 L·mol^–1^·min^–1^) was observed. One reason for the lower *D*_Gly_ of E1 could be that it hydrolyses and form the corresponding sulfonic acid which is actually E5 that showed very low *D*_Gly_ values (5.9%). The compounds E4 and F2–F5 showed low *D*_Gly_ values (<17%) in line with their *k*_Gly_ data (<0.11 L·mol^–1^·min^–1^, Table 1).

Comparing *D*_Gly_ with *D*_DPRA_ reveals that in particular the isocyanates and anhydrides show a substantially lower reactivity toward the DPRA lysine peptide (*D*_Gly_ = 100% vs 22% ≤ *D*_DPRA_ ≤ 83.5%; see Figure 1A) while the three acrylates F2–F4 are more reactive toward the lysine peptide (*D*_Gly_ < 16.6% vs *D*_DPRA_ > 86.5%).

**Figure 1:**
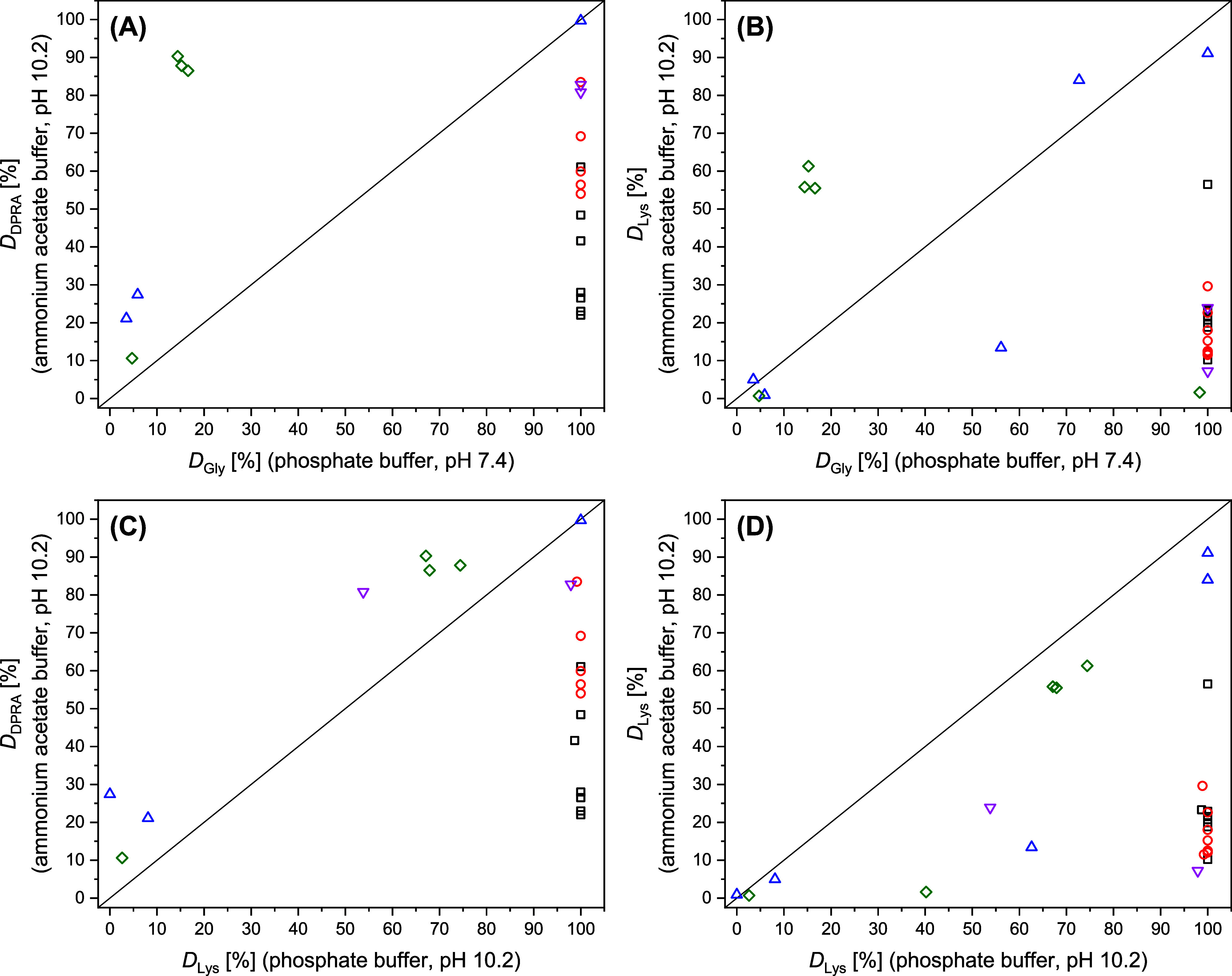
Comparison of the percentage depletion of the DPRA lysine peptide (*D*_DPRA_), Gly-pNA (*D*_Gly_), and Lys-pNA (*D*_Lys_) respectively, after reacting for 24 h with the iso­(thio)­cyanates [□ (black)], anhydrides [O (red)], S_N_Ar electrophiles [△ (blue)], acrylates [◇ (green)], and others [▽ (pink)].

A similar trend is observed when comparing *D*_Gly_ with *D*_Lys_ (pH 10.2, ammonium acetate buffer; Figure 1B). Here, isocyanates and anhydrides are also substantially less reactive toward Lys-pNA (*D*_Gly_ = 100% vs 10.2% ≤ *D*_Lys_ ≤ 56.5%). Moreover, 2,4-dinitrobenzenesulfonyl chloride (E1) moderately depletes Gly-pNA (*D*_Gly_ = 56.1%) but it is only slightly reactive toward Lys-pNA (*D*_Lys_ = 13.4%). Ethyl 2-cyanoacrylate (F1) and glutaraldehyde (G1) do not deplete Lys-pNA substantially in the ammonium acetate buffer (*D*_Lys_ < 7%) but both are highly reactive toward Gly-pNA (*D*_Gly_ > 98.3%). Interestingly, in phosphate buffer (pH 10.2) isocyanates and anhydrides almost entirely depleted Lys-pNA (*D*_Lys_ ≈ 100%), and thus show a substantially higher reactivity as compared to the DPRA lysine peptide (Figure 1C).

Finally, a comparison of the reactivity of the test compounds toward Lys-pNA in the two buffers (Figure 1D) again shows that the isocyanates, anhydrides, 2,4-dinitrobenzenesulfonyl chloride (E1), the cyanoacrylate (F1), glutaraldehyde (G1), and chloramine T (G2) are less reactive in the ammonium acetate buffer. All other test compounds show comparable Lys-pNA reactivity in both buffer setups.

In summary, the amino reactivity of some test compounds is substantially lower in ammonium acetate buffer compared to phosphate buffer. This may result from different stabilities of the test compounds in both buffers and will be analyzed in the next chapter.

### Degradation of the Test Compounds by the Aqueous Buffers Used

Water is the most abundant nucleophile in the aqueous media of the chemoassays used in the study. It may react with the test compounds (for proposed mechanisms see Scheme S4), and thus can disturb their availability for reacting with the amino target nucleophile.

Hence, the stability of the 27 test compounds in aqueous phosphate buffers (pH 7.4 and 10.2) and aqueous ammonium acetate buffer (pH 10.2) has been characterized in terms of half-lives (*t*_1/2_). The respective *t*_1/2_ values are listed in Table 3. For the two alkyl diisocyanates B1 and B2, ethyl 2-cyanoacrylate (F1), and glutaraldehyde (G1) *t*_1/2_ could not be determined because these compounds could not be detected by the HPLC UV–vis approach used (see above). Some test compounds were too rapidly depleted to record sufficient data points that can be used to calculate *t*_1/2_. For these, half-lives are expected to be smaller than 1 min (the first measurement was typically done after 1 min of incubation). This is indicated in Table 3 by *t*_1/2_ < 1 min.

**Table 3:** Stability of the 27 Test Compounds in Aqueous Phosphate Buffers (PPB, 80 mM) at pH 7.4 and 10.2 as well as in Ammonium Acetate Buffer (AAP, 100 mM) in Terms of Respective Half-Lives (*t*_1/2_)

compound	No	half-lives *t*_1/2_ ± s(*t*_1/2_) [min]		
		PPB pH 7.4	PPB pH 10.2	AAP pH 10.2
Isocyanates& Isothiocyanates				
2,4-toluene diisocyanate	A1	<1	<1	≪ 1
1,5-naphthaline diisocyanate	A2	3.6 ± 1.6	2.6 ± 1.1	≪ 1
2,6-toluene diisocyanate	A3	<1	<1	≪ 1
4,4′-methylene diphenyl diisocyanate	A4	5.9 ± 1.0	4.6 ± 0.3	≪ 1
phenyl isocyanate	A5	<1	<1	≪ 1
hexamethylene diisocyanate	B1	-	-	-
isophorone diisocyanate	B2	-	-	-
fluorescein isothiocyanate	B3	no depletion	3498 ± 236	8.21 ± 0.18
Anhydrides				
phthalic anhydride	C1	89.5 ± 4.1	<1	≪ 1
trimellitic anhydride	C2	110 ± 10	1	≪ 1
tetrachlorophthalic anhydride	C3	24.3 ± 0.6	<1	≪ 1
maleic anhydride	C4	35.3 ± 1.3	2.7	≪ 1
himic anhydride	C5	6.79 ± 0.13	1.30 ± 0.41	≪ 1
hexahydrophthalic anhydride	D1	4.36 ± 0.36	1.2	≪ 1
methyltetrahydrophthalic anhydride	D2	2.24 ± 0.03	<1	≪ 1
S_N_Ar electrophiles (trianzines and dinitrobenzenes)				
2,4-dinitrobenzenesulfonyl chloride	E1	<1	<1	≪ 1
2,4,6-trichloro-1,3,5-triazine	E2	1.14 ± 0.05	<1	≪ 1
1-fluoro-2,4-dinitrobenzene	E3	no depletion	615 ± 31	73.2 ± 9.7
1-chloro-2,4-dinitrobenzene	E4	no depletion	no depletion	no depletion
2,4-dinitrobenzenesulfonic acid	E5	no depletion	no depletion	no depletion
Acrylates				
ethyl 2-cyanoacrylate	F1			
methyl acrylate	F2	no depletion	943 ± 63	629 ± 48
butyl acrylate	F3	no depletion	4248 ± 1799	929 ± 59
ethyl acrylate	F4	no depletion	1348 ± 172	880 ± 119
methyl methacrylate	F5	no depletion	1878 ± 488	3063 ± 625
Others				
glutaraldehyde	G1	-	-	-
chloramine T	G2	no depletion	no depletion	58.5 ± 1.3

As can be seen in Table 3, the aromatic isocyanates A1–A5 (*t*_1/2_ ≤ 5.9 min), 2,4-dinitrobenzenesulfonyl chloride (E1, *t*_1/2_ < 1 min), and 2,4,6-trichloro-1,3,5-trianzine (E2, 1.14 min) are rapidly depleted in the phosphate buffer at pH 7.4. The anhydrides are slightly more stable, with *t*_1/2_ ranging from 2.24 min (D3) to 110 min (C2). For fluorescein isothiocyanate (B4), the dinitrobenzenes E3–E5, the acrylates F2–F5, and chloramine T (G2) no depletion could be observed, indicating that these compounds are stable under the given conditions.

In phosphate buffer at pH 10.2, all test compounds degraded faster as compared to pH 7.4 (see Table 3). Some examples are fluorescein isothiocyanate (B3), 1-fluoro-2,4-dinitrobenzene (E3), and the four acrylates F2–F5 (see Table 2). This can be traced back to a higher concentration of hydroxide ions (OH^–^) at this pH level (compared to pH 7.4). These OH^–^ ions hydrolyze the test compounds faster than water (H_2_O). The anhydrides showed a comparatively fast degradation within the first minute and a slower follow-up depletion afterward (see Figure S4, Supporting Information). The initial fast decrease is caused by higher OH^–^ concentration at pH 10.2. However, hydrolysis of anhydrides forms carboxylic acids (see (II) in Scheme S4) which dropped the pH level of the phosphate buffer from 10.2 down up to 8.8 (see Table S3). This pH level drop in turn can decrease the hydrolytic depletion of the anhydrides and may explain the slower follow-up depletion (see Figure S4, Supporting Information). Given a stable pH level of 10.2 one can expect a fast degradation of anhydrides as observed within the first minute. Hence, in Table 3, the half-lives of anhydrides in phosphate buffer at pH 10.2 refer to their degradation at the beginning of the hydrolytic reaction and have been approximated according to pseudo-1st order kinetics by using the peak area of the first measurement at pH 7.4 as a measure for the initial anhydride concentration and the first measurement at pH 10.2 as measure for the anhydride concentration after reacting for 1 min, keeping in mind that in both pH level setups the applied amount of anhydride was the same.

In ammonium acetate buffer, almost all test compounds are substantially faster depleted than in phosphate buffer (pH 10.2) although the pH level was the same in both setups (see last column of Table 3). The isocyanates A1–A5, all anhydrides, 2,4-dinitrobenzenesulfonyl chloride (E1), and 2,4,6-trichloro-1,3,5-triazine (E2) were fully depleted after 1 min (= first measurement). This is indicated by *t*_1/2_ ≪ 1 min in Table 3. The chromatographic signals of the acrylates F2–F4 decreased slightly faster in the ammonium acetate buffer (629 min ≤ *t*_1/2_ ≤ 3063 min) as compared to the phosphate buffer (943 min ≤ *t*_1/2_ ≤ 4248 min), and the two dinitrobenzenes E4 and E5, again, did not show any significant signal depletion. Overall, the comparatively fast degradation of the test compounds through the ammonium acetate buffer indicates further reaction besides hydrolysis that contributes to the loss of test compounds.

One possible route could be the direct reaction of ammonia with the test compounds. In an aqueous solution at pH 10.2, more than 90% of the total ammonia (*pK*_a_ = 9.25)^38^ occurs as neutral form (NH_3_) while less than 10% are protonated (NH_4_^+^). However, NH_3_ is a nucleophile and can react with the electrophilic test compounds. The potential mechanisms underlying these reactions are very similar to the reactions of the test compounds with amino groups (see Scheme 2) and are summarized in Scheme 3.

**Scheme 3:**
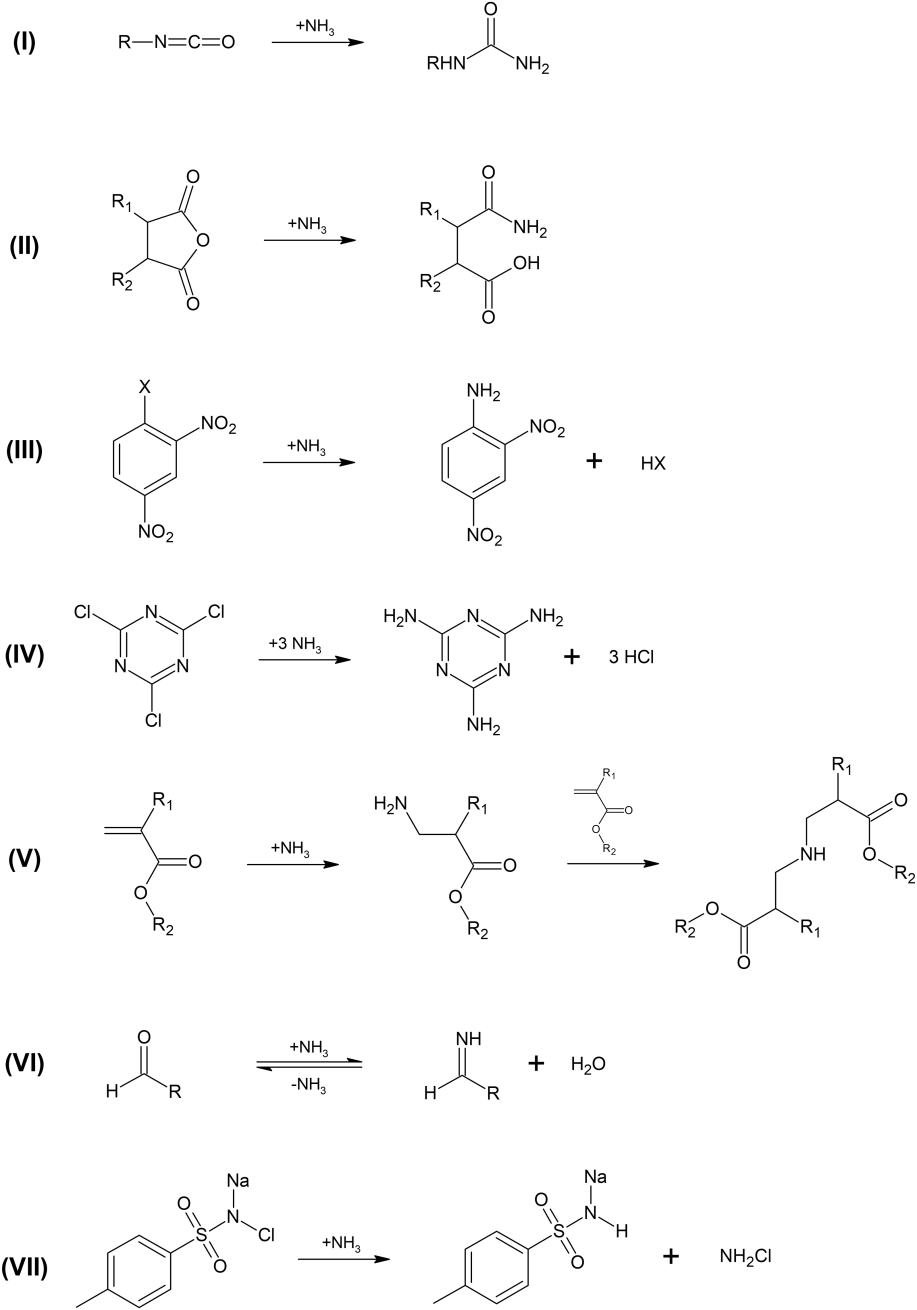
Mechanism Underlying the Reactions of Ammonia with Isocyanates (I), Anhydrides (II), Dinitrobenzenes (III), Trichlorotriazine (IV), Acrylates (V), Aldehydes (VI), and Chloramine T (VII)

To investigate this further, the chemical structures of adducts formed by the proposed reaction shown in Scheme 3 have been analyzed based on fragment patterns resulting from tandem MS product ion scans. The fragment spectra are shown in the Supporting Information in Figures S5–S45 together with proposed fragment structures. A formation of these ammonia adducts solely in the ionization source can be excluded due to use of ammonium-free eluents (see above). Hence, ammonia and ammonium ions are only present in the reaction mixtures and are chromatographically separated from the test compounds prior to ionization. For the diisocyanates A1–B3 addition of ammonia to one of the two isocyanate groups as shown by (I) in Scheme 3 could be confirmed (Figures S5–S11). In these monourea adducts, the second isocyanate group was hydrolytically converted into the respective amine (see (I) in Scheme S4). Moreover, mass spectrometric signals indicate the formation of double-urea adducts through the reaction of both isocyanate groups with ammonia (Figures S13–S18). For fluorescein isothiocyanate (B3) the respective thiourea adduct resulting from its reaction with NH_3_ was also observed (Figure S12). As proposed by (II) in Scheme 3, all anhydrides were converted into the respective amides through their reaction with NH_3_ (Figures S19–S25). In case of the four dinitrobenzenes E1, E3, E4, and E5 the reaction with ammonia formed 1-amino-2,4-dinitrobenzene ((III) in Scheme 3, Figures S26, S30–S32). Interestingly, ammonia adducts were also observed for E4 and E5, indicating that both react with NH_3_ but the reaction rates are too low to detect a substantial loss of these test compounds. Overall, the ammonia adducts of E1, E3, E4, and E5 further verify that they are formed in the reaction mixtures but not in the ionization source. In the case of the latter, ammonium would be added to the test compounds without replacing any groups at the aromatic ring. The so-formed adducts would show different molecular masses as compared to the adducts given in Figures S26 and S30–S32. In case of 2,4,6-trichloro-1,3,5-triazine (E2) the three reaction products demonstrate the stepwise substitution of the three chlorine by NH_3_ ((IV) in Scheme 3, Figures S27–S29). For the reactions of the acrylates (F1–F5) with ammonia mass spectrometric signals indicate the formation of the Michael adduct ((V) in Scheme 3, Figures S33–S37). These Michael adducts are primary amines and react further with a second acrylate to form ammonia-acrylate double adducts (Figures S38–S42). Glutaraldehyde formed imines with NH_3_ ((VI) in Scheme 3; Figures S43 and S44) while chloramine T did not form a direct ammonia adduct. It reacted with NH_3_ by replacing chlorine by hydrogen ((VII) in Scheme 3, Figure S45).

To sum up, the analysis of the adduct patterns resulting from the reactions of the test compounds with ammonia confirmed our suspicion that ammonia as a nucleophile depletes the test compounds, and thus can hamper the assessment of their amino reactivity in the DPRA.

### Amino Reactivity vs Respiratory Sensitization Potential

As outlined in the introduction, previous studies showed that respiratory sensitizers could not be sufficiently well identified and discriminated from dermal allergens based on their amino reactivity preference in the DPRA. This conclusion was mainly drawn because isocyanates, well-known as respiratory sensitizers, did not show a high amino reactivity as observed for other respiratory allergens such as anhydrides.^4,18–20^ Another critical factor hampering the identification of respiratory sensitizers by alternative methods (chemoassay, in vitro, in silico) is the lack of a proven reference test system that can clearly identify respiratory allergens and allows for discriminating between immunologically (true respiratory sensitizers) and nonimmunologically (asthmagens) acting candidates.^12–14^ Hence, information on the respiratory sensitization potential of chemicals typically results from clinical observation or workplace studies but cannot provide sufficient information whether the underlying mechanism is based on an immunological response or not.^12^ Here, acrylates and methacrylates are prominent examples. In earlier studies, they were often classified as respiratory sensitizers^2,3,20^ which is underpinned by regulatory proposals developed by the ECHA.^58–61^ However, recent studies have raised doubt on the respiratory sensitization potential of acrylates and methacrylates^62,63^ and current reference lists of true (immunologically acting) respiratory sensitizers do not contain acrylates or methacrylates but cyanoacrylates.^4,5,41^

As described above and summarized by Table S1, the 27 test compounds have been classified as either potential respiratory sensitizers or nonsensitizers. Figure 2 compares this categorization with the reactivity of the test compounds toward the DPRA lysine peptide (*D*_DPRA_), Gly-pNA (log *k*_Gly_, *D*_Gly_), and Lys-pNA (*D*_Lys_, phosphate buffer, pH 10.2).

**Figure 2:**
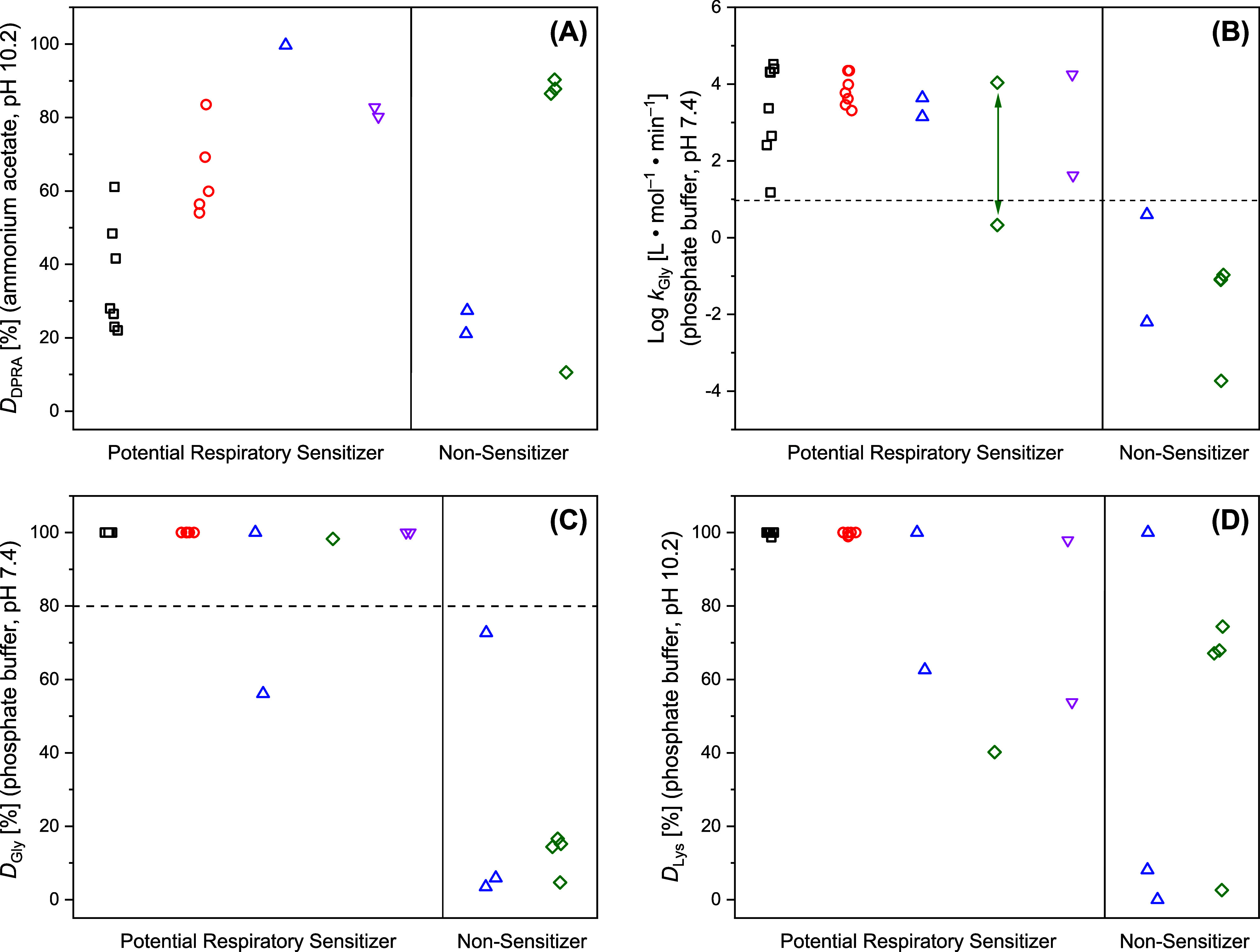
Comparison of the respiratory sensitization potential of the investigated iso­(thio)­cyanates [□ (black)], anhydrides [O (red)], S_N_Ar electrophiles [△ (blue)], acrylates [◇ (green)], and others [▽ (pink)] with their reactivity toward the DPRA lysine peptide (*D*_DPRA_ (A)), Gly-pNA (log *k*_Gly_ (B) and *D*_Gly_ (C)), and Lys-pNA (*D*_Lys_ (D)) respectively. The green arrow in graph (B) reflect the varying reactivity of ethyl 2-cyanoacrylate (F1). The dashed lines indicate tentative Gly-pNA reactivity thresholds for the identification of potential respiratory sensitizers.

As shown in Figure 2A, the DPRA lysine reactivity profile of the test compounds cannot discriminate between potential respiratory sensitizers and nonsensitizing candidates. In both groups, *D*_DPRA_ values range between 10% and 100%, and the nonsensitizing acrylates (F2–F4) are even more reactive than isocyanates and anhydrides (*D*_DPRA_: ≥86.5% vs ≤ 83.5%, see also Table 2) due to the comparatively strong depletion of isocyanates and anhydrides by the ammonium acetate buffer (see Table 3).

Using log *k*_Gly_ as a measure of amino reactivity, indeed, allows for the discrimination between the two groups (Figure 2B). Almost all potential respiratory sensitizers show a log *k*_Gly_ ≥ 1.18 while all nonsensitizers are less reactive (log *k*_Gly_ ≤ 0.6). In contrast to the DPRA setup (Figure 2A), all isocyanates and anhydrides show high amino reactivities (log *k*_Gly_ ≥ 2.41) in the Gly-pNA chemoassay because they are more stable in the phosphate buffer as compared to the DPRA ammonium acetate buffer. Also the less reactive isothiocyanate (B3, log *k*_Gly_ ≥ 1.18) is significantly more reactive than the most reactive nonsensitizer 1-fluoro-2,4-dinitrobenzene (E3) with log *k*_Gly_ = 0.6. For ethyl 2-cyanoacrylate (F1), we added both log *k*_Gly_ values (0.35 and 4.02) to Figure 2B to indicate that its amino reactivity can vary over orders of magnitude depending on the concentration of water in the surrounding medium. In water-poor regions of lung proteins like their hydrophobic surface or pockets that are shielded from bulk water, the amino reactivity of F1 might be increased because the fraction that is bound to water is decreased. Hence, the high log *k*_Gly_ value of F1 shown in Figure 2B illustrates its potential for an increased amino reactivity and indicates the unique amino reactivity of F1 as compared to the nonsensitizing but iso-reactive 1-fluoro-2,4-dinitrobenzene (E3). Moreover, in water-rich regions of the lungs water blocks the reactive Michael acceptor group of F1 from being attacked by other cellular protective nucleophiles such as glutathione. Hence, the cyanoacrylate might be conserved for reacting with amino groups for a longer period of time (as compared to fast eliminated electrophiles) which may also contribute to its sensitization potential. Addressing this scenario of varying and conserved amino reactivity by routine reactivity testing is challenging because kinetic chemoassays are typically conducted in aqueous media within a few minutes or hours.^44,64^ Water-poor chemoassay setups using acetonitrile as reaction medium^65^ can overcome limitations of water-based variants but imply of course additional testing effort.

As can be seen in Figure 2C, Gly-pNA was almost entirely depleted by many potential respiratory sensitizers (see *D*_Gly_ values in Table 2), including also ethyl 2-cyanoacrylate (F1, *D*_Gly_ = 98.3%). This indicates that the DPRA-like setup with the longer incubation time of 24 h and the 50-fold excess of F1 over Gly-pNA reflects the varying and conserved amino reactivity of F1 better than *k*_Gly_. 2,4-dinitrobenzenesulfonyl chloride (E1) shows a substantially lower depletion (*D*_Gly_ = 56.1%) which is even lower than *D*_Gly_ of the most reactive nonsensitizer E3 (*D*_Gly_ = 72.7%) and can be explained by its low half-live even in phosphate buffer at pH 7.4 (*t*_1/2_ < 1 min, see Table 3). Hence, E1 is fast depleted in the DPRA-type setup and given the 50-fold excess of E1 over Gly-pNA, already after six half-lives (< 6 min) E1 cannot entirely deplete Gly-pNA anymore because it is no longer in excess over the nucleophile. In addition, its Gly-pNA reactivity in terms of *k*_Gly_ (4387 L·mol^–1^·min^–1^) is not high enough to fully deplete Gly-pNA within the first 6 min of incubation which may explain the observed lower *D*_Gly_ value of E1 (as compared to the other potential respiratory sensitizers). The iso-reactive himic anhydride (*k*_Gly_ = 4126 L·mol^–1^·min^–1^), for example, fully depletes Gly-pNA in the DPRA-like setup (*D*_Gly_ = 100%) because it is substantially more stable in phosphate buffer at pH 7.4 (*t*_1/2_ = 6.79 min). Anyway, one has to keep in mind that E1 is not listed as a potential respiratory sensitizer (see Table S1) and only showed respective characteristics in vitro.^43^

Finally, no clear discrimination between potential respiratory sensitizers and nonsensitizers can be achieved when applying *D*_Lys_ (phosphate buffer, pH 10.2) as a measure for their amino reactivity (Figure 2D). Here, sensitizing isocyanates and anhydrides show similar *D*_Lys_ values (≥98.7%) as the nonsensitizer E3 (*D*_Lys_ = 100%). Moreover, the potential respiratory sensitizers E1 and F1 are even less reactive (*D*_Lys_: 62.6% and 40.2%) than the nonsensitizing acrylates F2–F4 (*D*_Lys_ ≥ 67.1%), probably because the latter are more stable at pH 10.2 (see Table 3).

To sum up, the identification of potential respiratory sensitizers based on their amino reactivity in the DPRA can be substantially hampered by the degradation of highly amino-reactive test compounds through the ammonium acetate buffer to be used according to the DPRA OECD protocol.^31^ To improve this protocol, the reactivity of nonamino buffers based on boron or carbonates toward highly reactive electrophiles could be evaluated in future studies in order to identify suitable alternatives to the ammonium acetate buffer at pH 10.2. However, one should keep in mind that this pH level does not reflect physiological conditions well. In contrast, the Gly-pNA chemoassay applying a phosphate buffer at a physiological pH level of 7.4 allows for a substantial better discrimination between potential respiratory sensitizers and nonsensitizers due to improved test compound stability. However, further research is needed to transfer the results of our mechanistic study into a reactivity assay that is suitable to screen sets of chemicals for potential respiratory sensitizers. The DPRA-like setup with Gly-pNA appears promising for this purpose because of its standardized setup, its comparatively low experimental effort, and the good prognostic identification of true respiratory sensitizers (see Figure 2C). To address also further key events along the AOP of true respiratory sensitization, respective immunologic in vitro assays^13,21–23^ could be combined with amino reactivity assay data to assess the respiratory sensitization potential based on weight of evidence approaches.^66^

Finally, the reactivity of electrophiles toward the thiol group of cysteine also has been discussed as a potential driver of the respiratory sensitization potential.^4,18,19^ In the oxygen-rich respiratory tract, thiol groups occur in its oxidized form (R–S–S–R) and the recently proposed reaction of the R–S–S–R group with nucleophilic sensitizers^67^ may come into play, which of course needs to be further investigated by future chemoassay analyses.

## Conclusion

The amino reactivity of organic electrophiles appears as a useful measure for the identification of true respiratory sensitizers causing pulmonary diseases via hypersensitive immune responses. Characterization of the amino reactivity with the OECD-accepted DPRA, however, can be hampered by the degradation of the test compounds through the ammonium acetate buffer, confounding the envisaged reaction with the target nucleophile. This in particular holds for highly reactive isocyanates and anhydrates but should be also investigated for other amino-reactive compound classes such as aldehydes or 1,2-dicarbonyls. The Gly-pNA chemoassay overcomes this issue by using phosphate buffer as reaction medium, and thus may serve as a nonanimal screening method as part of an AOP-driven integrated approach for testing and assessment (IATA) of the respiratory sensitization potential in the frameworks of occupational safety and the global harmonized system of labeling and packaging of chemicals (GHS CLP). Therefore, future studies with the Gly-pNA chemoassay may focus on testing further known true respiratory sensitizers as well as nonsensitizers and pure dermal sensitizers in order to examine to what extent *k*_Gly_ and *D*_Gly_ (alone or in combination) provide thresholds for a prospective assessment of the respiratory sensitization potential. Finally, the Gly-pNA may also improve the amino reactivity-based assessment of the dermal sensitization potential of chemicals under REACH because in the DPRA their amino reactivity might be underestimated due to the nonintentional reaction with the ammonium acetate buffer. This, of course, needs to be analyzed in detail by future studies.

## Supplementary Material



## Notes

One table informing about the classification of the test compounds as either potential respiratory sensitizers or nonsensitizers; three figures illustrating the determination of *k*_Gly_; one table summarizing the experimental conditions of the DPRA-type setups; one table summarizing the settings of the UV vis detector used to analyze the degradation of the test compounds in the aqueous reaction media; one scheme proposing the mechanism for the reaction of the sulfonyl chloride group of compound E1 with an amino group; one scheme proposing the mechanism for the reactions of ethyl 2-cyanoacrylate (F1) with water and Gly-pNA; experimental details for the determination of the dimensionless equilibrium constant (*K*) for the reaction of ethyl 2-cyanoacrylate (F1) with water; one scheme proposing the mechanisms for the reaction of the test compounds with water; one table summarizing the pH levels in the DPRA-like setup using the phosphate buffer at pH 10.2; one figure illustrating the different degradation patterns of the anhydrides in phosphate buffers at pH 7.4 and 10.2, respectively; 40 figures showing the product ion spectra and fragment structures of the adducts formed by the reactions of the test compounds with ammonia; experimental details for the determination of the dimensionless equilibrium constant (*K*) for the reaction of ethyl 2-cyanoacrylate (F1) with water (PDF)
