# Supplementary material for: Refining the Amino Reactivity-Based Identification of Respiratory Sensitizers
Source: Chem Res Toxicol. 2025 May 29;38(6):1046–60. doi: 10.1021/acs.chemrestox.4c00545 (PMC12175168; doi:10.1021/acs.chemrestox.4c00545)
Supplement: Supplementary file 1 [file tx4c00545_si_001.pdf]

Refining the Amino Reactivity-Based Identification of Respiratory Sensitizers

Martin Simoneit[†], Helene Langer[†], Nadin Ulrich[†], and Alexander Böhme[†,*]

[†]UFZ Department of Exposure Science, Helmholtz Centre for Environmental Research, Permoserstraße 15, 04318 Leipzig, Germany.

Contents: One table informing about the classification of the test compounds as either potential respiratory sensitizers or non-sensitizers; three figures illustrating the determination of k_{Gly}; one table summarizing the experimental conditions of the DPRA-type setups; one table summarizing the settings of the UV vis detector used to analyze the degradation of the test compounds in the aqueous reaction media; one scheme proposing the mechanism for the reaction of the sulfonyl chloride group of compound E1 with an amino group; one scheme proposing the mechanism for the reactions of ethyl 2-cyanoacrylate (F1) with water and Gly-pNA; experimental details for the determination of the dimensionless equilibrium constant (*K*) for the reaction of ethyl 2-cyanoacrylate (F1) with water; one scheme proposing the mechanisms for the reaction of the test compounds with water; one table summarizing the pH levels in the DPRA-like setup using the phosphate buffer at pH 10.2; one figure illustrating the different degradation patterns of the anhydrides in phosphate buffers at pH 7.4 and 10.2, respectively; 40 figures showing the product ion spectra and fragment structures of the adducts formed by the reactions of the test compounds with ammonia; experimental details for the determination of the dimensionless equilibrium constant (*K*) for the reaction of ethyl 2-cyanoacrylate (F1) with water.

Table S1. Information on the classification of the 27 test compounds as either potential respiratory sensitizers or non-sensitizers.

Compound	CAS-No.	No.	Classification	Rational for Classification
		Isocyanates & Isothiocyanates		
2,4-Toluene diisocyanate	584-84-9	A1	potential respiratory sensitizer	True resp. sensitizer in Krutz *et al.* based on clinical evidence in humans along with an immunological assessment.[1] Highest evidence in Sadekar *et al.* based on epidemiological studies and clinical reports.[2] Identified as clinical resp. sensitizers in Ponder *et al.*[3]
1,5-Naphthaline diisocyanate	3173-72-6	A2	potential respiratory sensitizer	High evidence in Sadekar *et al.* based on epidemiological studies and clinical reports.[2]
2,6-Toluene diisocyanate	91-08-7	A3	potential respiratory sensitizer	True resp. sensitizer in Krutz *et al.* based on clinical evidence in humans along with an immunological assessment.[1] Highest evidence in Sadekar *et al.* based on epidemiological studies and clinical reports.[2] Identified as clinical resp. sensitizers in Ponder *et al.*[3]
4,4′-Methylene diphenyl diisocyanate	101-68-8	A4	potential respiratory sensitizer	Highest evidence in Sadekar *et al.* based on epidemiological studies and clinical reports.[2] Identified as clinical resp. sensitizers in Ponder *et al.*[3]
Phenyl isocyanate	103-71-9	A5	potential respiratory sensitizer	Identified as respiratory sensitizer in Karol *et al.*[4] Isocyanates, in general, are considered as respiratory sensitizers in the reference lists.[1–3]

Hexamethylene diisocyanate	822-06-0	B1	potential respiratory sensitizer	True resp. sensitizer in Krutz *et al.* based on clinical evidence in humans along with an immunological assessment.[1] Highest evidence in Sadekar *et al.* based on epidemiological studies and clinical reports.[2] Identified as clinical resp. sensitizers in Ponder *et al.*[3]
Isophorone diisocyanate	4098-71-9	B2	potential respiratory sensitizer	True resp. sensitizer in Krutz *et al.* based on clinical evidence in humans along with an immunological assessment.[1]
Fluorescein isothiocyanate	3326-32-7	B3	potential respiratory sensitizer	Reference respiratory sensitizer in Hopkins *et al.*[5]
		Anhydrides		
Phthalic anhydride	85-44-9	C1	potential respiratory sensitizer	True resp. sensitizer in Krutz *et al.* based on clinical evidence in humans along with an immunological assessment.[1] Identified as clinical resp. sensitizers in Ponder *et al.*[3]
Trimellitic anhydride	552-30-7	C2	potential respiratory sensitizer	True resp. sensitizer in Krutz *et al.* based on clinical evidence in humans along with an immunological assessment.[1] Highest evidence in Sadekar *et al.* based on epidemiological studies and clinical reports.[2] Identified as clinical resp. sensitizers in Ponder *et al.*[3]
Tetrachlorophthalic anhydride	117-08-8	C3	potential respiratory sensitizer	True resp. sensitizer in Krutz *et al.* based on clinical evidence in humans along with an immunological assessment.[1] High evidence in Sadekar *et al.* based on epidemiological studies and clinical reports.[2]
Maleic anhydride	108-31-6	C4	potential respiratory sensitizer	True resp. sensitizer in Krutz *et al.* based on clinical evidence in humans along with an immunological assessment.[1]

				Highest evidence in Sadekar *et al.* based on epidemiological studies and clinical reports.[2]
Himic anhydride	2746-19-2	C5	potential respiratory sensitizer	Highest evidence in Sadekar *et al.* based on epidemiological studies and clinical reports.[2]
Hexahydrophthalic anhydride	85-42-7	D1	potential respiratory sensitizer	Highest evidence in Sadekar *et al.* based on epidemiological studies and clinical reports.[2]<br>Identified as clinical resp. sensitizers in Ponder *et al.*[3]
Methyltetrahydrophthalic anhydride	19438-64-3	D2	potential respiratory sensitizer	High evidence in Sadekar *et al.* based on epidemiological studies and clinical reports.[2]<br>Identified as clinical resp. sensitizers in Ponder *et al.*[3]
		S_NAr electrophiles (trianzines & dinitrobenzenes)		
2,4-Dinitrobenzenesulfonyl chloride	1656-44-6	E1	potential respiratory sensitizer	Similar behavior in vitro as other potential respiratory sensitizers in Hopkins *et al.*[5]
2,4,6-Trichloro-1,3,5-triazine	108-77-0	E2	potential respiratory sensitizer	True resp. sensitizer in Krutz *et al.* based on clinical evidence in humans along with an immunological assessment.[1]
1-Fluoro-2,4-dinitrobenzene	70-34-8	E3	non-sensitizer	Reference respiratory non-sensitizers in Hopkins *et al.*[5]<br>Not listed as potential respiratory sensitizer in one of the reference lists.[1–3]
1-Chloro-2,4-dinitrobenzene	97-00-7	E4	non-sensitizer	Reference respiratory non-sensitizers in Hopkins *et al.*[5]<br>Not listed as potential respiratory sensitizer in one of the reference lists.[1–3]
2,4-Dinitrobenzenesulfonic acid	89-02-1	E5	non-sensitizer	Similar structure as E3 and E4, which were used as reference respiratory non-sensitizers in Hopkins *et al.*[5]

				Not listed as potential respiratory sensitizer in one of the reference lists.[1–3]
		Acrylates		
Ethyl 2-cyanoacrylate	7085-85-0	F1	potential respiratory sensitizer	Highest evidence in Sadekar *et al.* based on epidemiological studies and clinical reports.[2]
Methyl acrylate	96-33-3	F2	non-sensitizer	No evidence for aliphatic acrylates in Sadekar *et al.* based on epidemiological studies and clinical reports.[2]
Butyl acrylate	141-32-2	F3	non-sensitizer	
Ethyl acrylate	140-88-5	F4	non-sensitizer	
Methyl methacrylate[b]	80-62-6	F5	non-sensitizer	Low evidence in Sadekar *et al.* based on epidemiological studies and clinical reports.[2]<br>Reported doubts on the respiratory sensitization potential of methacrylates.[6,7]
		Others		
Glutharaldehyde	111-30-8	G1	potential respiratory sensitizer	True resp. sensitizer in Krutz *et al.* based on clinical evidence in humans along with an immunological assessment.[1]<br>High evidence in Sadekar *et al.* based on epidemiological studies and clinical reports.[2]<br>Identified as clinical resp. sensitizers in Ponder *et al.*[3]
Chloramine T	127-65-1	G2	potential respiratory sensitizer	True resp. sensitizer in Krutz *et al.* based on clinical evidence in humans along with an immunological assessment.[1]<br>Highest evidence in Sadekar et al. based on epidemiological studies and clinical reports.[2]<br>Identified as clinical resp. sensitizers in Ponder *et al.*[3]

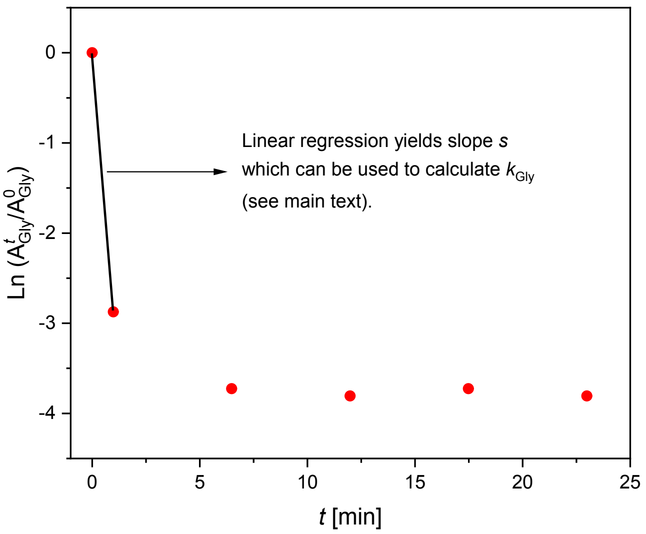

Figure S1. Experimental data for the reaction of Gly-pNA with 2,6-toluene diisocyanate (A3) to illustrate determination of the minimum reactivity in terms of k_{Gly} for the highly reactive isocyanates A1-A5, the anhydrides C1-C4, 2,4-dinitrobenzenesulfonyl chloride (E1), and 1,3,5-richloro-2,4,6-triazine (E2).

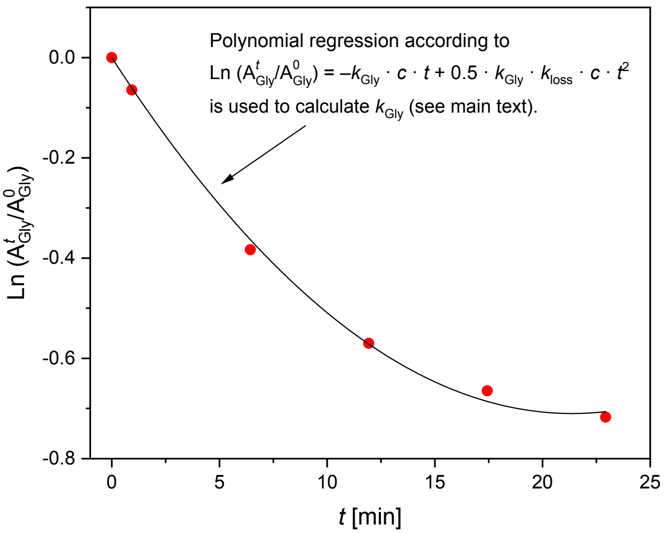

Figure S2. Experimental data for the reaction of Gly-pNA with hexamethylene diisocyanate (B1) to illustrate the determination of k_{Gly} for the isocyanates B1 and B2, the anhydrides C5-D2, and methyl methacrylate (F5). These compounds showed a curved trend for the depletion of Gly-pNA.

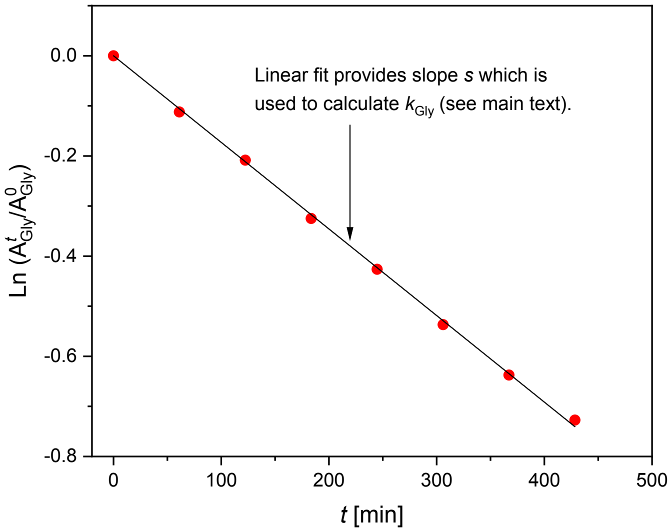

Figure S3. Experimental data for the reaction of Gly-pNA with methyl acrylate (F2) to illustrate the determination of k_{Gly} for the compounds B3, E3-E5, F1-F4, and G2.

Summary of experimental conditions of the DPRA-like setups used to determine D_{Gly}, D_{Lys}, and D_{DPRA} data in Table 2 (main text).

Table S2. Overview of experimental conditions for the DPRA-like setups used to determine 24 h percentage depletion rates for the reaction of the test compounds with Gly-pNA, Lys-pNA, and the DPRA lysine peptide (literature data).

	**Gly-pNA** (D_{Gly})	**Lys-pNA** (D_{Lys})	**DPRA lysine peptide** (D_{DPRA})
Nucleophile concentration	0.03 mM	0.03 mM	0.5 mM
Excess of test compound	50-fold	50-fold	50-fold
pH value of used buffer	7.4	10.2	10.2
Used buffers	phosphate	phosphate & ammonium acetate	ammonium acetate
Vol-% of acetonitrile	26.7 %	26.7 %	25 %
Temperature	25°C	25 °C	25 °C

Summary of the settings of the UV vis detector used to analyze the degradation of the test compounds in the aqueous reaction media.

Table S3. Overview on chromatographic method and the wavelengths used for analyzing the stability of the test compounds in the aqueous reaction media.

Compounds	No	Chrom. Method	Wavelength
Isocyanates & Isothiocyanates			
2,4-Toluene diisocyanate	A1	gradient	270 nm
1,5-Naphthaline diisocyanate	A2	gradient	270 nm
2,6-Toluene diisocyanate	A3	gradient	270 nm
4,4′-Methylene diphenyl diisocyanate	A4	gradient	270 nm
Phenyl isocyanate	B1	gradient	270 nm
Hexamethylene diisocyanate	B2	-	-
Isophorone diisocyanate	B3	-	-
Fluorescein isothiocyanate	B4	gradient	290 nm
Anhydrides			
Phthalic anhydride	C1	gradient	270 nm
Trimellitic anhydride	C2	gradient	270 nm
Tetrachlorophthalic anhydride	C3	isocratic	254 nm
Maleic anhydride	C4	isocratic	254 nm
Himic anhydride	D1	isocratic	235 nm
Hexahydrophthalic anhydride	D2	isocratic	235 nm
Methyltetrahydrophthalic anhydride	D3	isocratic	235 nm
S_NAr electrophiles (trianzines & dinitrobenzenes)			
2,4-Dinitrobenzenesulfonyl chloride	E1	isocratic	270 nm
2,4,6-Trichloro-1,3,5-triazine	E2	isocratic	270 nm
1-Fluoro-2,4-dinitrobenzene	E3	isocratic	290 nm
1-Chloro-2,4-dinitrobenzene	E4	isocratic	290 nm
2,4-Dinitrobenzenesulfonic acid	E5	isocratic	290 nm
Acrylates			
Ethyl 2-cyanoacrylate	F1	isocratic	254 nm
Methyl acrylate	F2	isocratic	254 nm
Butyl acrylate	F3	isocratic	254 nm
Ethyl acrylate	F4	isocratic	254 nm
Methyl methacrylate	F5	isocratic	254 nm
Others			
Glutaraldehyde	G1	-	-
Chloramine T	G2	gradient	254 nm

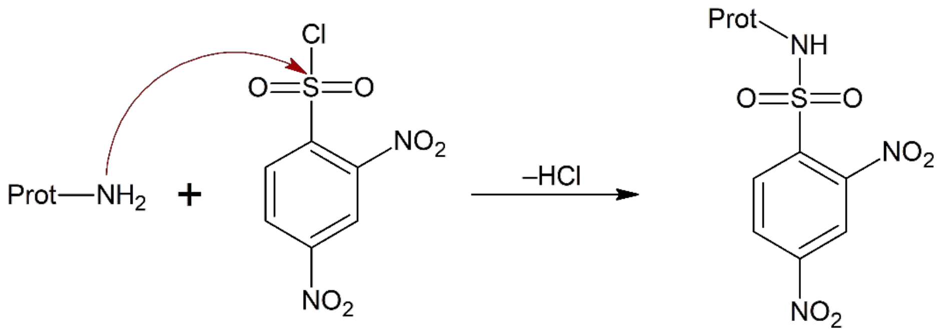

Scheme S1. Proposed mechanism for the Hinsberg-type reaction triggered through the nucleophilic attack of an amino group at the sulfonyl chloride group of E1.

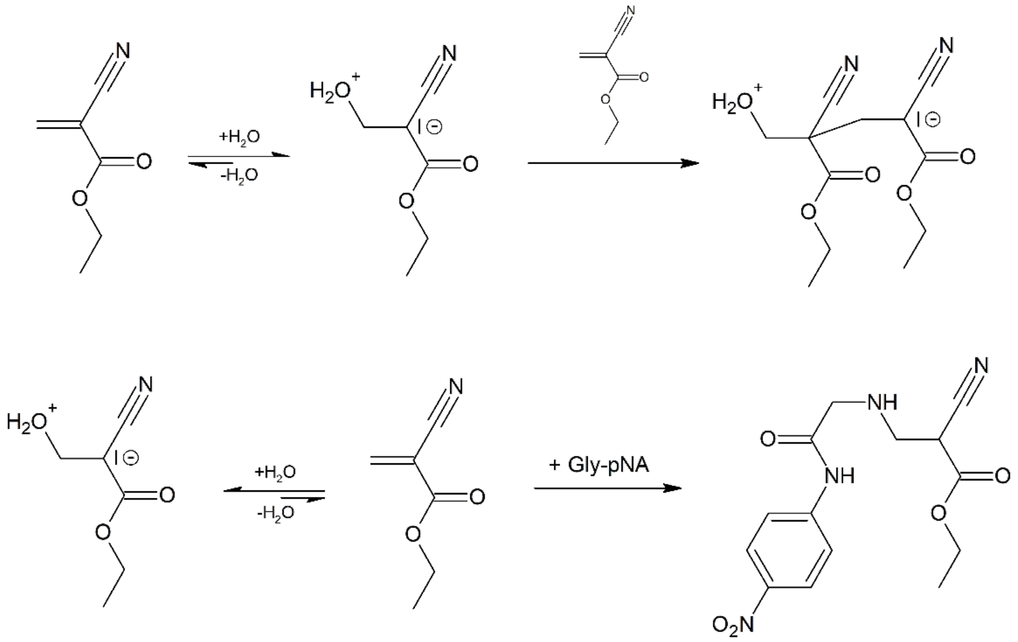

Scheme S2. Proposed reactions of ethyl 2-cyanoacrylate (F1) with water (top) and Gly-pNA (bottom).

Determination of the dimensionless equilibrium constant (*K*) for the reaction of ethyl 2-cyanoacrylate (F1) with water.

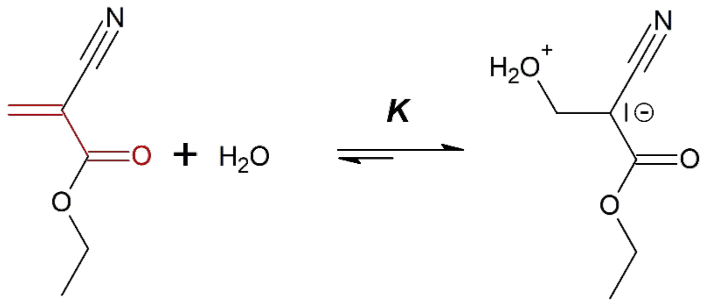

Scheme S3. Reaction of ethyl 2-cyanoacrylate (F1) with water. The structural feature highlighted in red indicates the Michael acceptor group (C=C–C=O) used for the UV based quantification of the free amount of F1 at λ = 230 nm.

To determine *K*, the UV absorbance caused by the Michael acceptor group (C=C–C=O) of F1 was measured in acetonitrile (ACN) using a SpectraMax384^{Plus} (Molecular Devices) without and in the presence of water. As reaction batches, glass cuvettes (1 cm thickness, 3 mL total volume) were used. First, the absorbance of F1 in ACN was measured at 230 nm. Afterwards water was added to the cuvette and after 10 minutes the absorbance at 230 nm was recorded again several time to ensure that equilibrium was reached. This experiment was repeated four times with different concentrations of F1 and water, which is summarized in Table S4 together with the determined values of *K*.

Table S4. Overview on the experimental details used to determine *K* for the reaction of ethyl 2-cyanoacrylate (ECA) with water.

V_{ACN} [mL]	c_{ECA} [mol/L]	c_{water} [mol/L]	$A_{t=0}$ (230 nm)	A_{eq} (230 nm)	*K*
2	0.002	0.028	1.785	0.398	126
2	0.002	0.042	2.470	0.450	108
2	0.003	0.028	2.962	0.534	164
2	0.003	0.042	3.075	0.67	86.3

Based on the data given in Table S4, *K* has been calculated according to Eq. S1:

$$K = \frac{c_{ECA} - c_{ECA} \cdot A_{t=0}/A_{eq}}{c_{ECA} \cdot A_{t=0}/A_{eq} \cdot c_{water}} \quad \text{(S1)}$$

Here and in Table S4, c_{ECA} and c_{water} are the used concentrations of ethyl 2-cyanoacrylate and water, respectively, and $A_{t=0}$ and A_{eq} denote the measured absorbance at the beginning (t = 0) and after the equilibrium shown in Scheme S3 was reached.

(I)

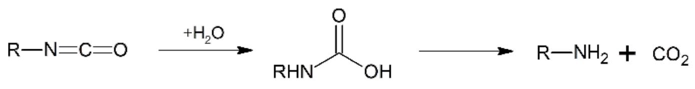

(II)

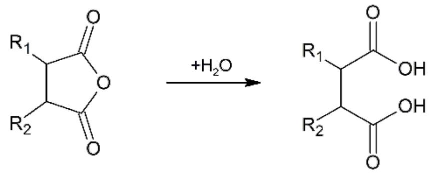

(III)

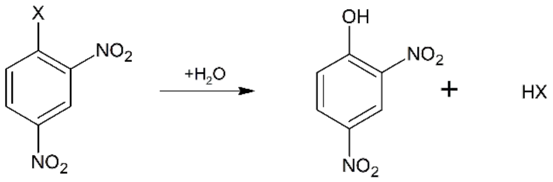

(IV)

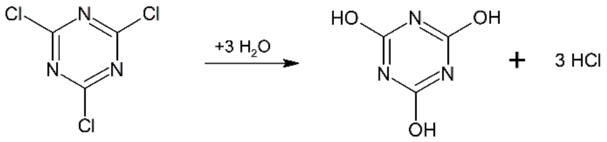

(V)

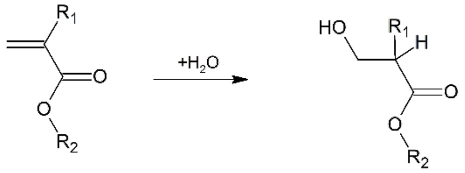

(VI)

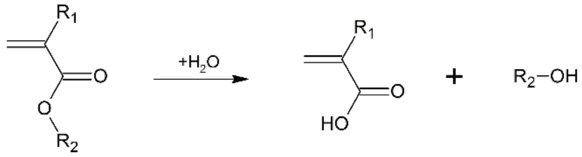

(VII)

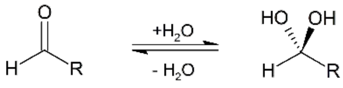

(VIII)

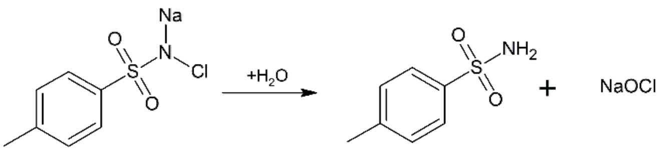

Scheme S4. Proposed mechanisms underlying the reaction of water with isocyanates (I), anhydrides (II), dinitrobenzenes (III), trichlorotriazines (IV), acrylates ((V) and (IV)), aldehydes (VII), and chloramine T (VIII).

Overview on pH levels in the DPRA-like setup using the phosphate buffer at pH 10.2

Table S5. Overview on the pH levels for the reactions of Gly-pNA with the 27 test compounds in phosphate buffer at pH 10.2 directly after adding the test compound (*t* = 0 h) as well as after 24 h incubation (*t* = 24 h).

Compounds	No	pH (*t* = 0 h)	pH (*t* = 24 h)
Isocyanates & Isothiocyanates			
2,4-Toluene diisocyanate	A1	9.9	9.5
1,5-Naphthaline diisocyanate	A2	10.2	9.5
2,6-Toluene diisocyanate	A3	9.7	9.4
4,4′-Methylene diphenyl diisocyanate	A4	9.8	9.6
Phenyl isocyanate	B1	10.2	10.0
Hexamethylene diisocyanate	B2	10.2	10.0
Isophorone diisocyanate	B3	10.4	10.1
Fluorescein isothiocyanate	B4	9.2	9.1
Anhydrides			
Phthalic anhydride	C1	9.3	9.2
Trimellitic anhydride	C2	8.9	8.8
Tetrachlorophthalic anhydride	C3	9.3	9.1
Maleic anhydride	C4	9.5	9.2
Himic anhydride	D1	9.3	9.0
Hexahydrophthalic anhydride	D2	9.4	9.2
Methyltetrahydrophthalic anhydride	D3	9.3	9.1
S_NAr electrophiles (trianzines & dinitrobenzenes)			
2,4-Dinitrobenzenesulfonyl chloride	E1	9.1	9.1
2,4,6-Trichloro-1,3,5-triazine	E2	9.1	9.1
1-Fluoro-2,4-dinitrobenzene	E3	10.5	9.8
1-Chloro-2,4-dinitrobenzene	E4	10.5	10.5
2,4-Dinitrobenzenesulfonic acid	E5	10.0	10.0
Acrylates			
Ethyl 2-cyanoacrylate	F1	9.8	9.3
Methyl acrylate	F2	10.5	10.4
Butyl acrylate	F3	10.5	10.5
Ethyl acrylate	F4	10.5	10.4
Methyl methacrylate	F5	10.5	10.5
Others			
Glutaraldehyde	G1	10.3	10.4
Chloramine T	G2	10.4	10.3

Peak areas for the chromatographic signals of tetrachlorophthalic anhydride (C3) to illustrate the different degradation patterns of the anhydrides in phosphate buffers at pH 7.4 and 10.2, respectively.

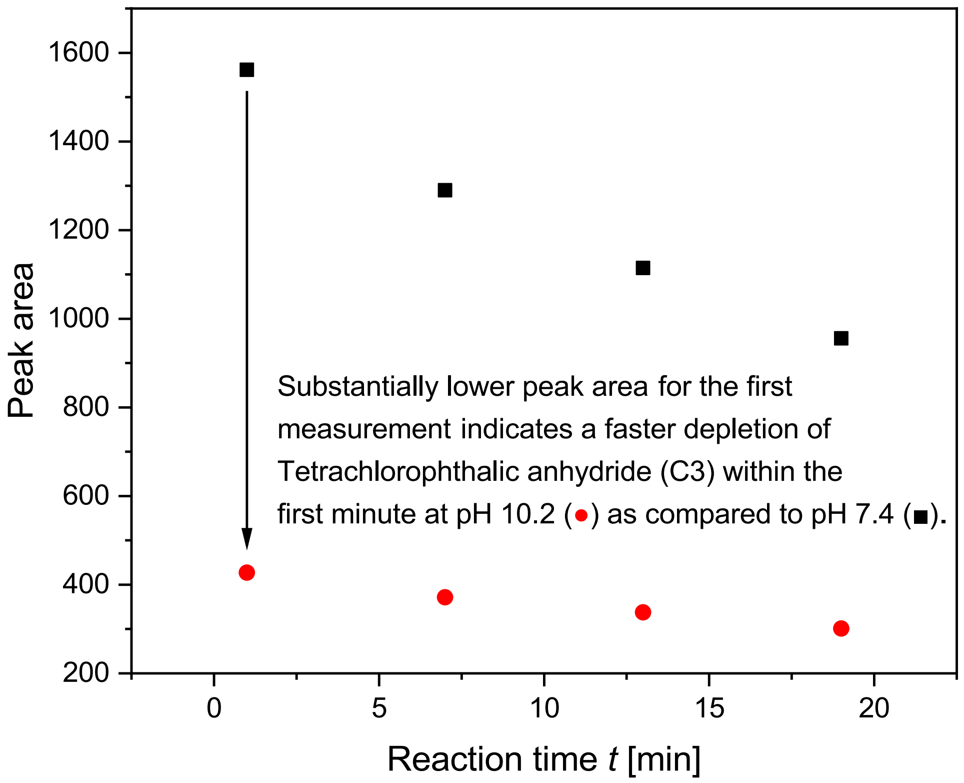

Figure S4. Comparison of the peak areas below the chromatographic signals of tetrachlorophthalic anhydride (C3) for its hydrolytic degradation in aqueous phosphate buffers at pH 7.4 (■) and pH 10.2 (●).

Product ion scan-derived fragmentation patterns of adducts formed by the reactions of ammonia with the test compounds.

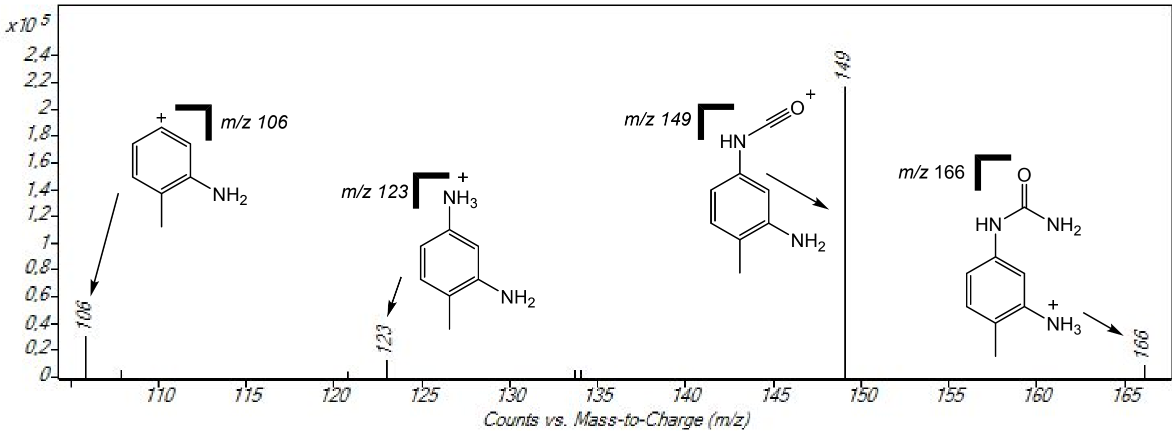

Figure S5. Product ion spectrum and proposed fragment structures of the mono-urea adduct with *m/z* 166 Da formed by the reaction of 2,4-toluene diisocyanate (A1) and ammonia (NH_3).

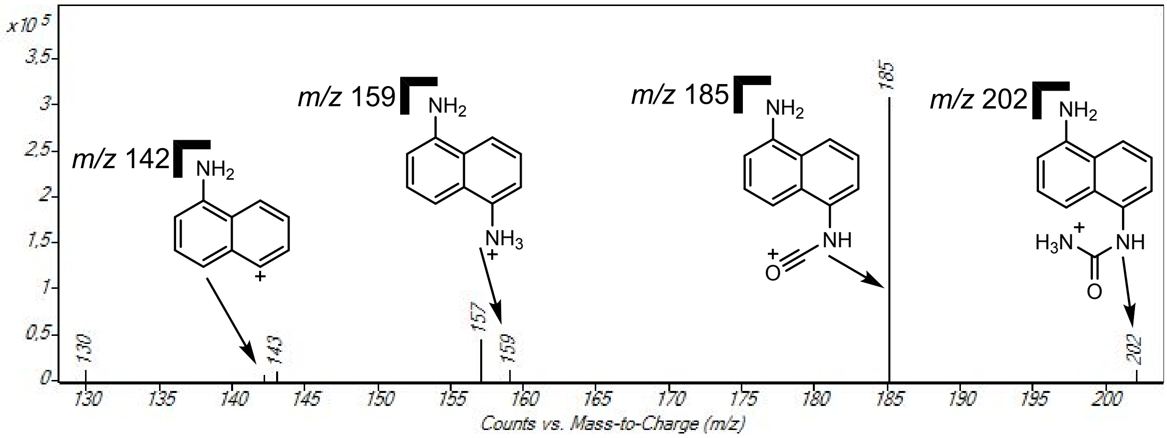

Figure S6. Product ion spectrum and proposed fragment structures of the mono-urea adduct with *m/z* 202 Da formed by the reaction of 1,5-naphthalene diisocyanate (A2) and ammonia (NH_3).

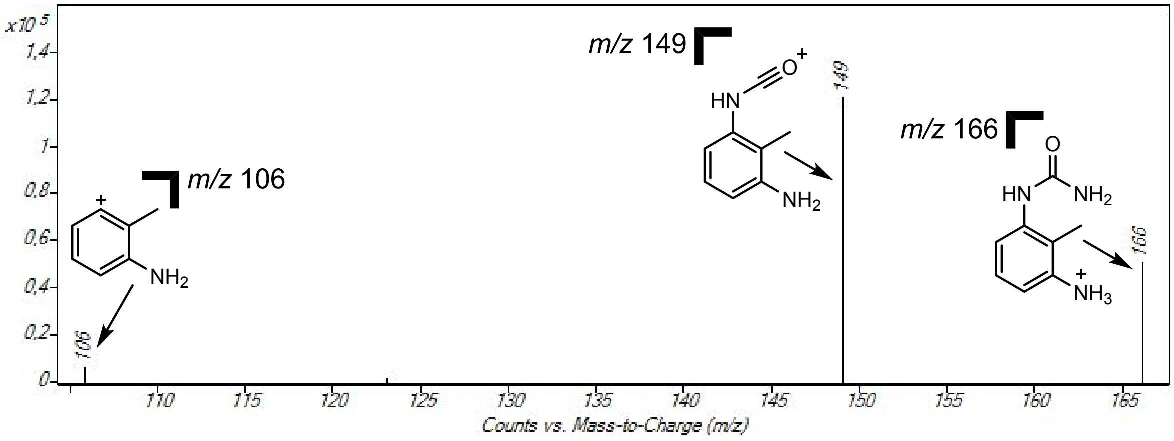

Figure S7. Product ion spectrum and proposed fragment structures of the mono-urea adduct with *m/z* 166 Da formed by the reaction of 2,6-toluene diisocyanate (A3) and ammonia (NH_3).

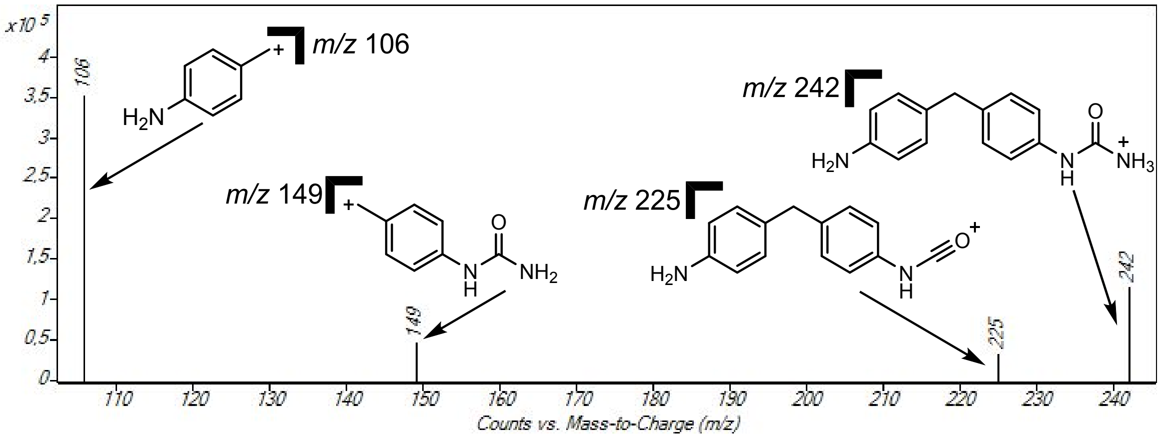

Figure S8. Product ion spectrum and proposed fragment structures of the mono-urea adduct with *m/z* 242 Da formed by the reaction of 4,4-diphenylmethane diisocyanate (A4) and ammonia (NH_3).

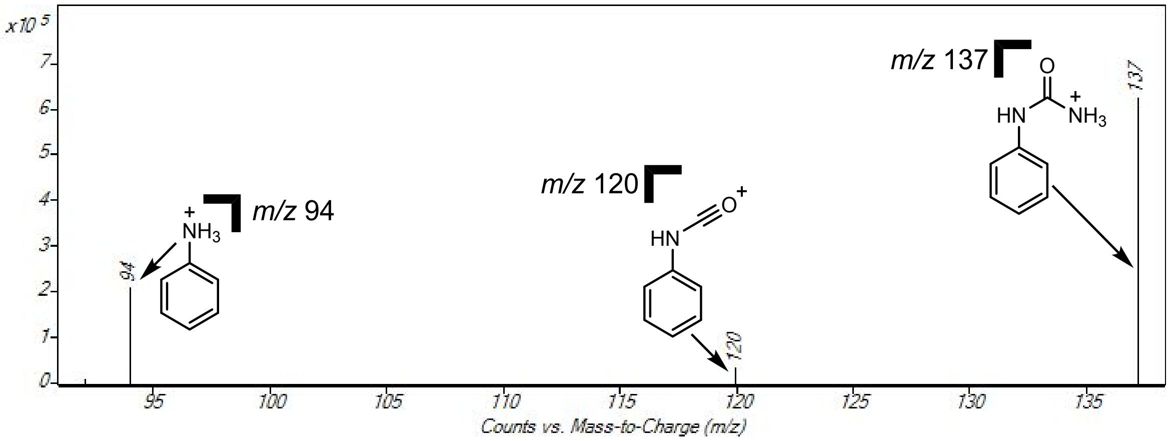

Figure S9. Product ion spectrum and proposed fragment structures of the mono-urea adduct with *m/z* 137 Da formed by the reaction of phenyl isocyanate (A5) and ammonia (NH_3).

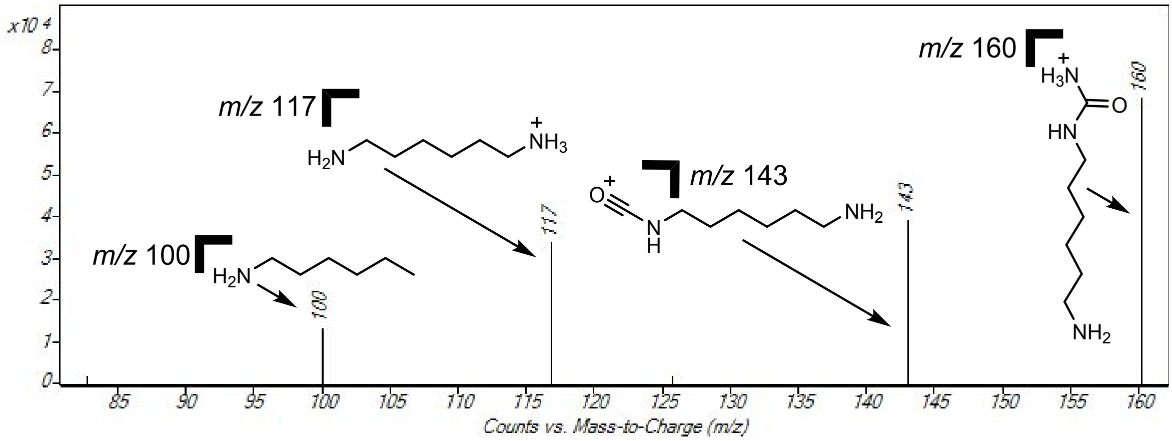

Figure S10. Product ion spectrum and proposed fragment structures of the mono-urea adduct with *m/z* 160 Da formed by the reaction of hexamethylene diisocyanate (B1) and ammonia (NH_3).

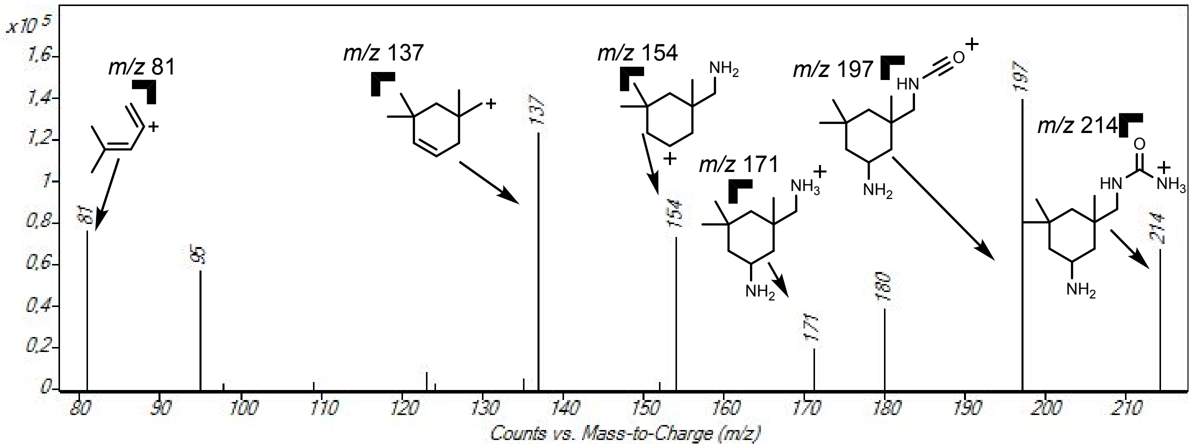

Figure S11. Product ion spectrum and proposed fragment structures of the mono-urea adduct with *m/z* 214 Da formed by the reaction of isophorone diisocyanate (B2) and ammonia (NH_3).

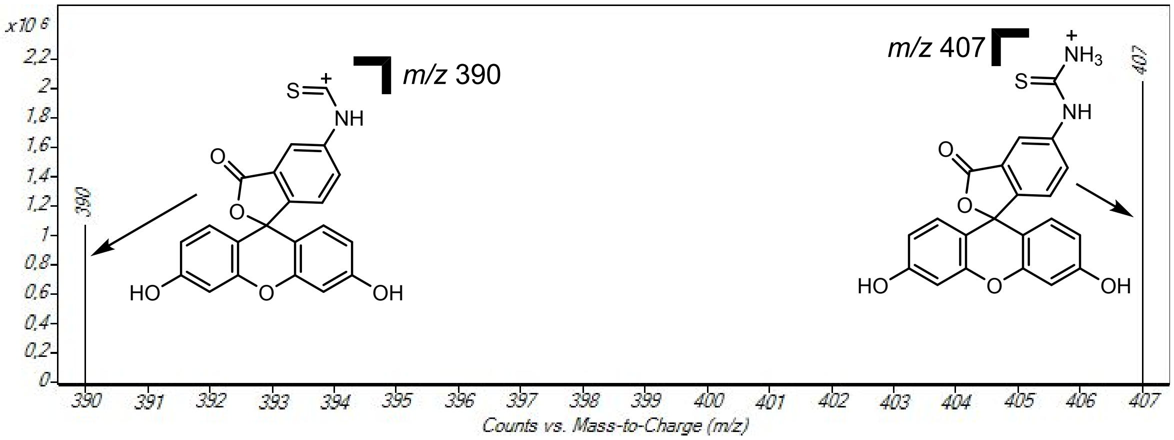

Figure S12. Product ion spectrum and proposed fragment structures of the thiourea adduct with *m/z* 407 Da formed by the reaction of fluorescein isothiocyanate (B3) and ammonia (NH_3).

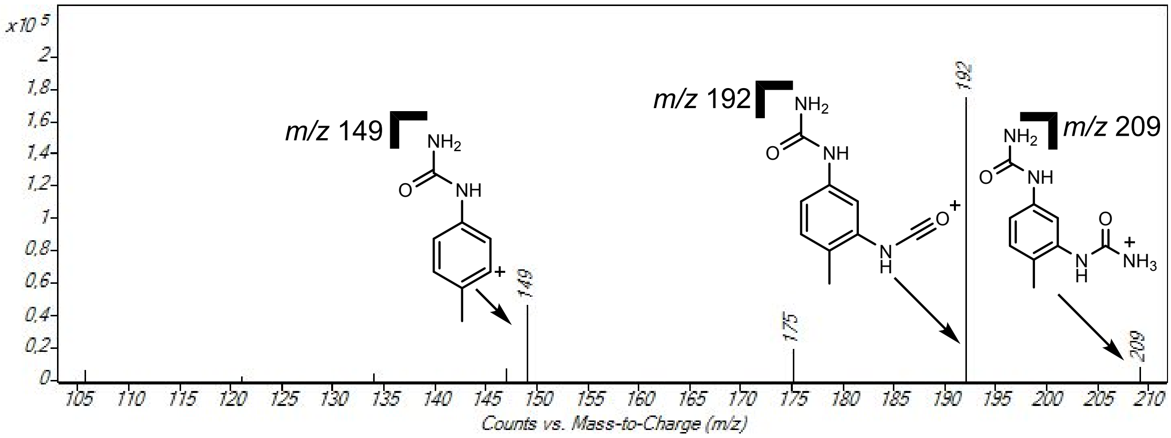

Figure S13. Product ion spectrum and proposed fragment structures of the di-urea adduct with *m/z* 209 Da formed by the reaction of 2,4-toluene diisocyanate (A1) and two molecules of ammonia (NH_3).

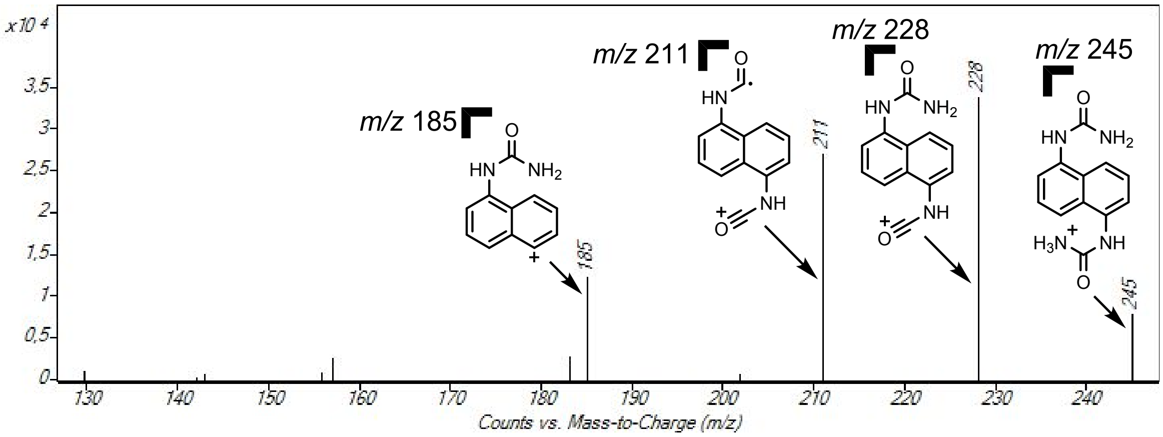

Figure S14. Product ion spectrum and proposed fragment structures of the di-urea adduct with *m/z* 245 Da formed by the reaction of 1,5-naphthalene diisocyanate (A2) and two molecules of ammonia (NH_3).

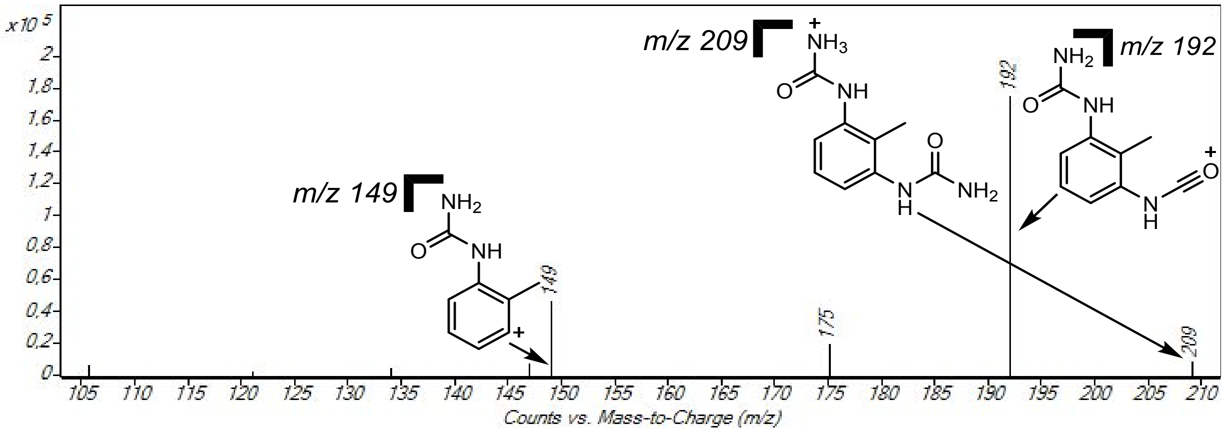

Figure S15. Product ion spectrum and proposed fragment structures of the di-urea adduct with *m/z* 209 Da formed by the reaction of 2,6-toluene diisocyanate (A3) and two molecules of ammonia (NH_3).

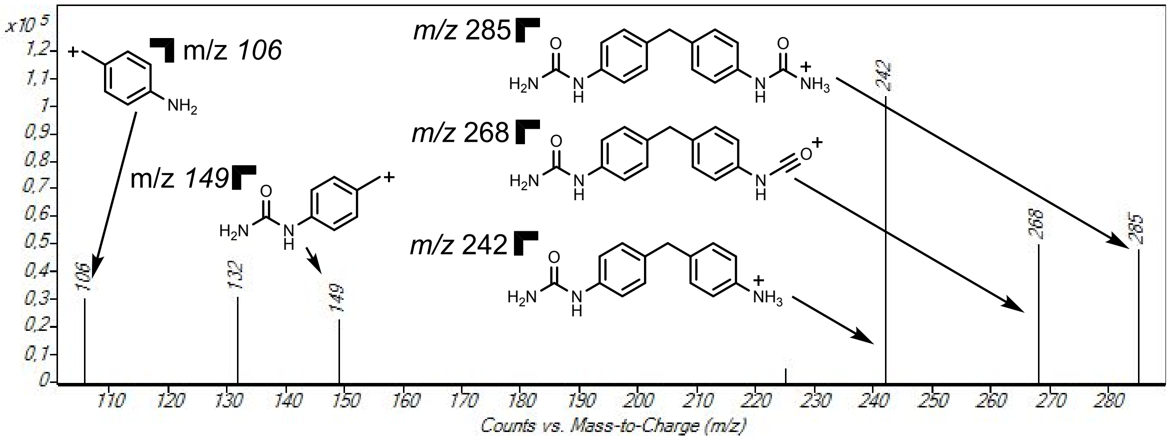

Figure S16. Product ion spectrum and proposed fragment structures of the di-urea adduct with *m/z* 285 Da formed by the reaction of 4,4-diphenylmethane diisocyanate (A4) and two molecules of ammonia (NH_3).

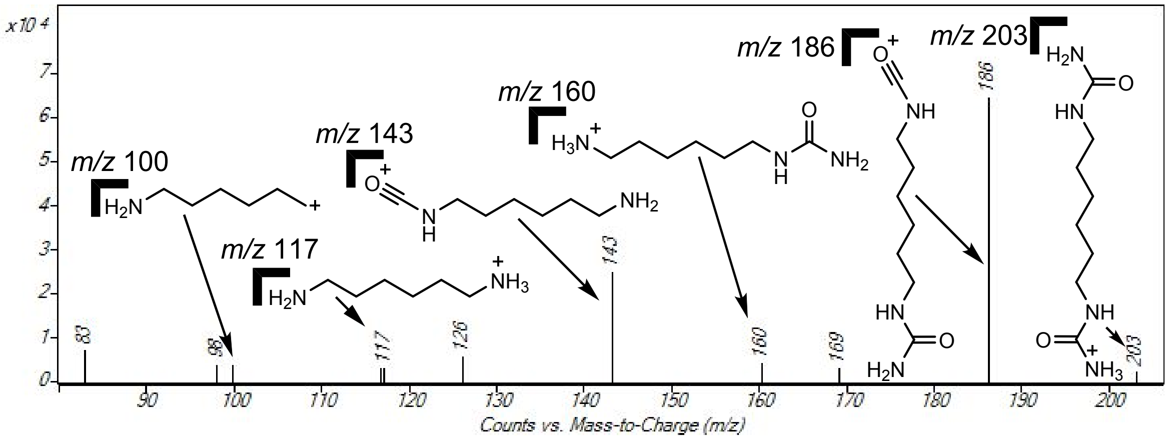

Figure S17. Product ion spectrum and proposed fragment structures of the di-urea adduct with *m/z* 203 Da formed by the reaction of hexamethylene diisocyanate (B1) and ammonia (NH_3).

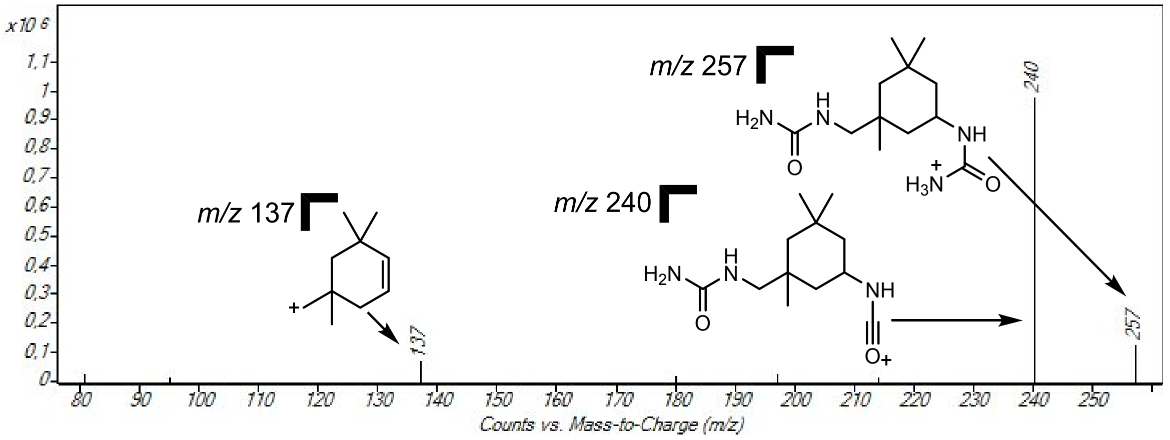

Figure S18. Product ion spectrum and proposed fragment structures of the di-urea adduct with *m/z* 257 Da formed by reaction of isophorone diisocyanate (B2) and two molecules of ammonia (NH_3).

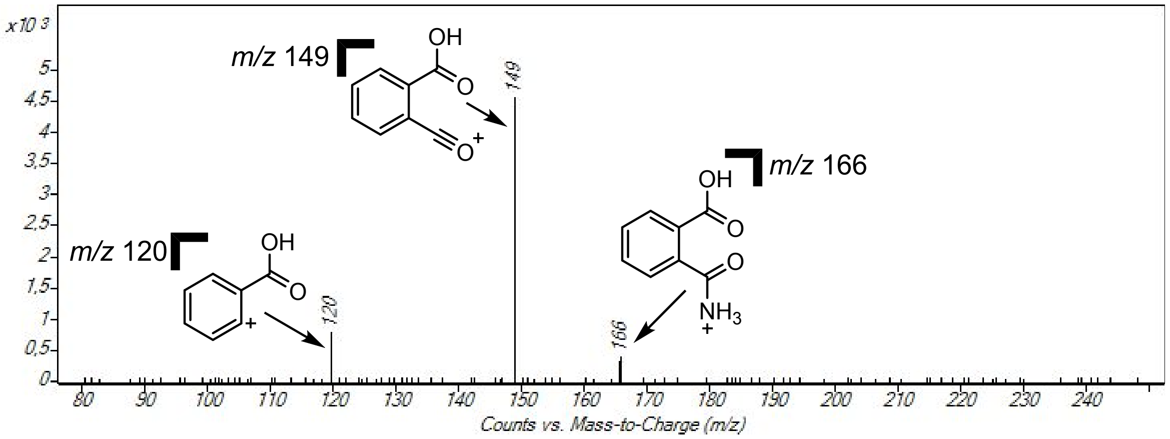

Figure S19. Product ion spectrum and proposed fragment structures of the amide adduct with *m/z* 166 Da formed by the reaction of phthalic anhydride (C1) and ammonia (NH_3).

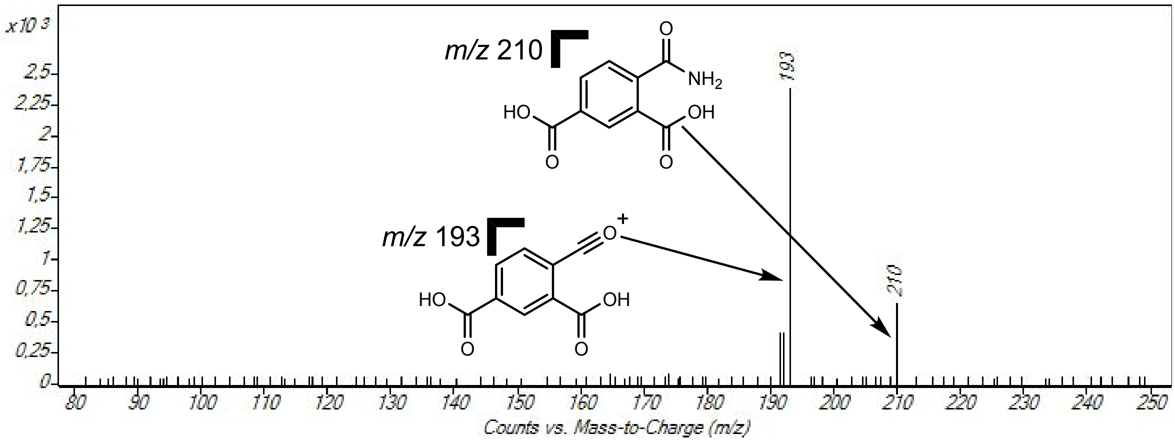

Figure S20. Product ion spectrum and proposed fragment structures of the amide adduct with *m/z* 210 Da formed by the reaction of trimellitic anhydride (C2) and ammonia (NH_3).

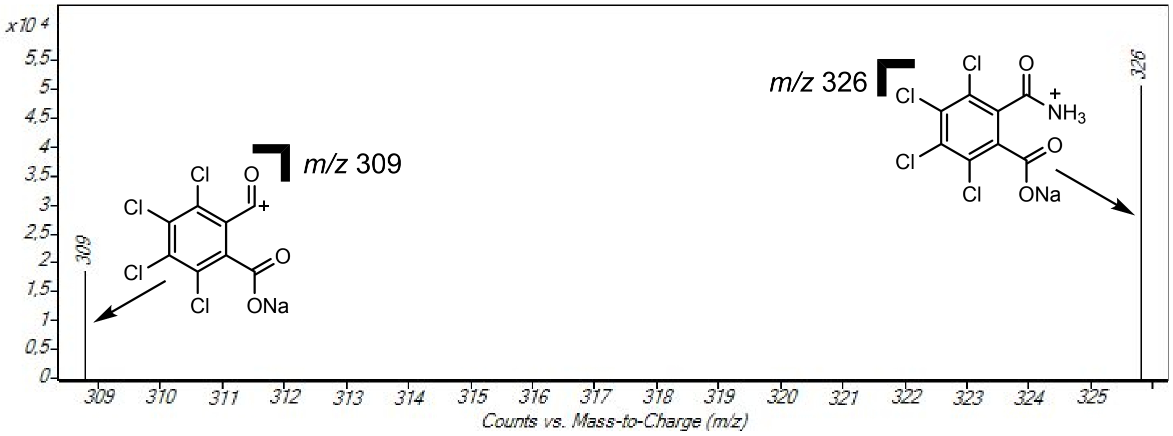

Figure S21. Product ion spectrum and proposed fragment structures of the amide adduct with *m/z* 326 Da (sodium adduct) formed by the reaction of tetrachlorophthalic anhydride (C3) and ammonia (NH_3).

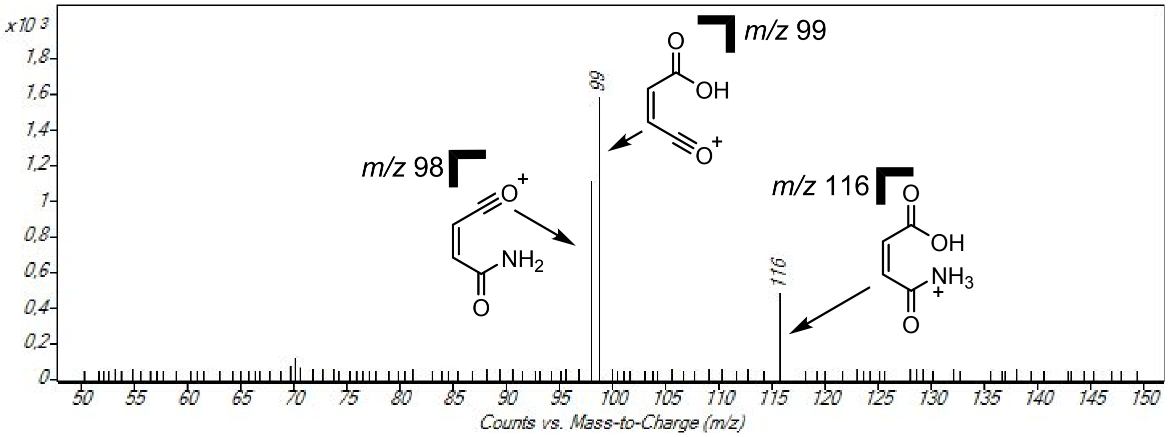

Figure S22. Product ion spectrum and proposed fragment structures of the amide adduct with *m/z* 116 Da formed by the reaction of maleic anhydride (C4) and ammonia (NH_3).

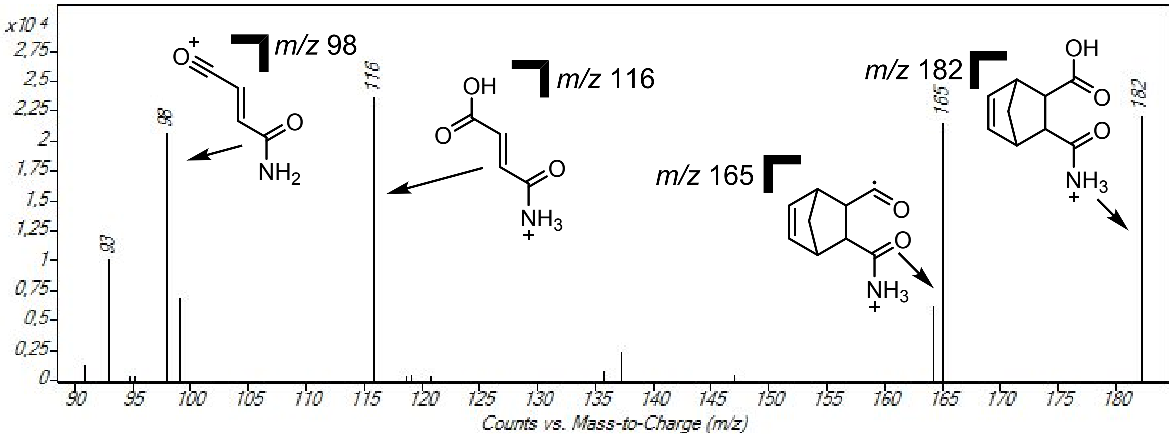

Figure S23. Product ion spectrum and proposed fragment structures of the amide adduct with *m/z* 182 Da formed by the reaction of himic anhydride (C5) and ammonia (NH_3).

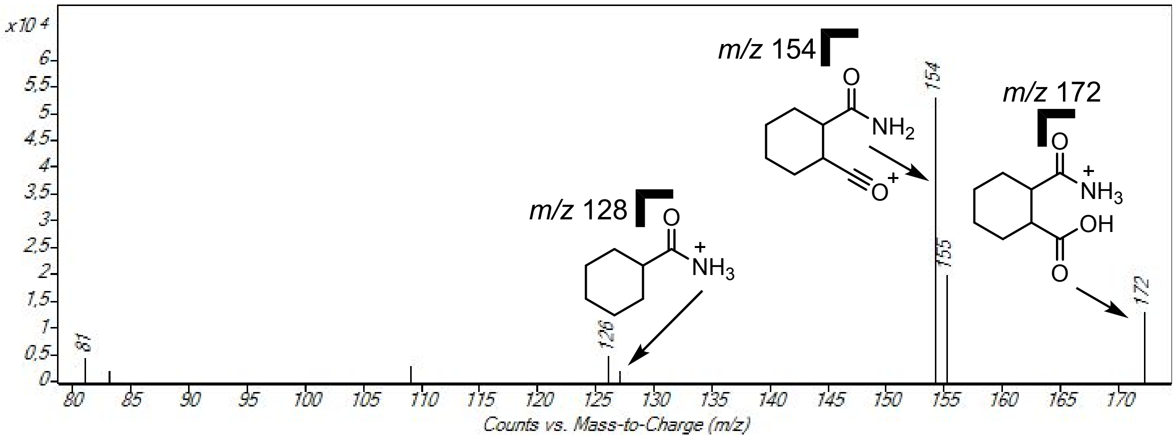

Figure S24. Product ion spectrum and proposed fragment structures of the amide adduct with *m/z* 172 Da formed by the reaction of hexahydrophthalic anhydride (D1) and ammonia (NH_3).

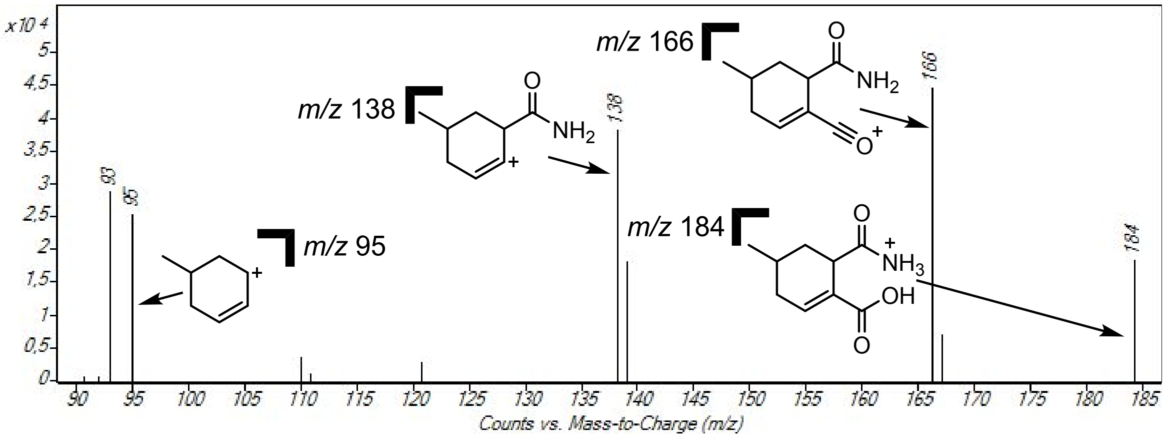

Figure S25. Product ion spectrum and proposed fragment structures of the amide adduct with *m/z* 184 Da formed by the reaction of methyltetrahydrophthalic anhydride (D2) and ammonia (NH_3).

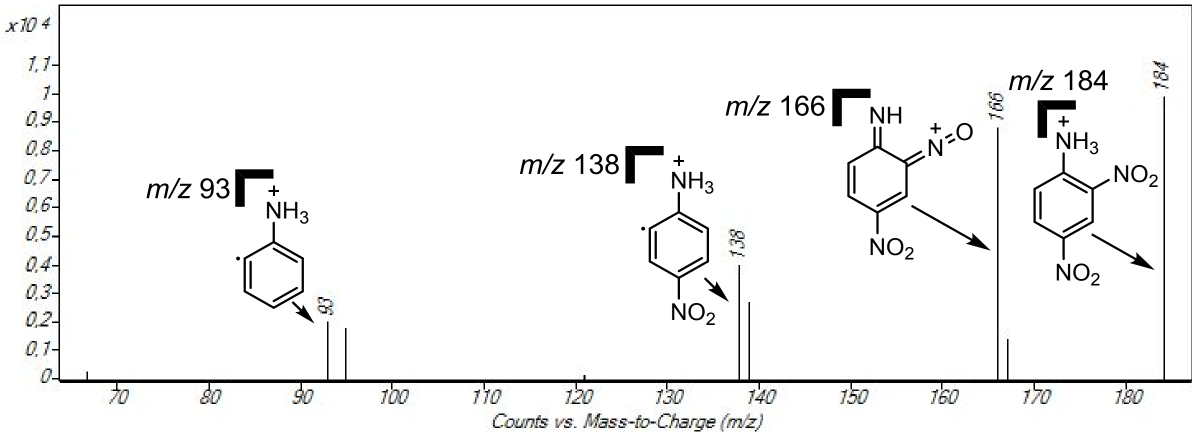

Figure S26. Product ion spectrum and proposed fragment structures of the 1-amino-2,4-dinitrobenzene adduct with *m/z* 184 Da formed by the reaction of 2,4-dnitrobenzenesulfonyl chloride (E1) and ammonia (NH_3).

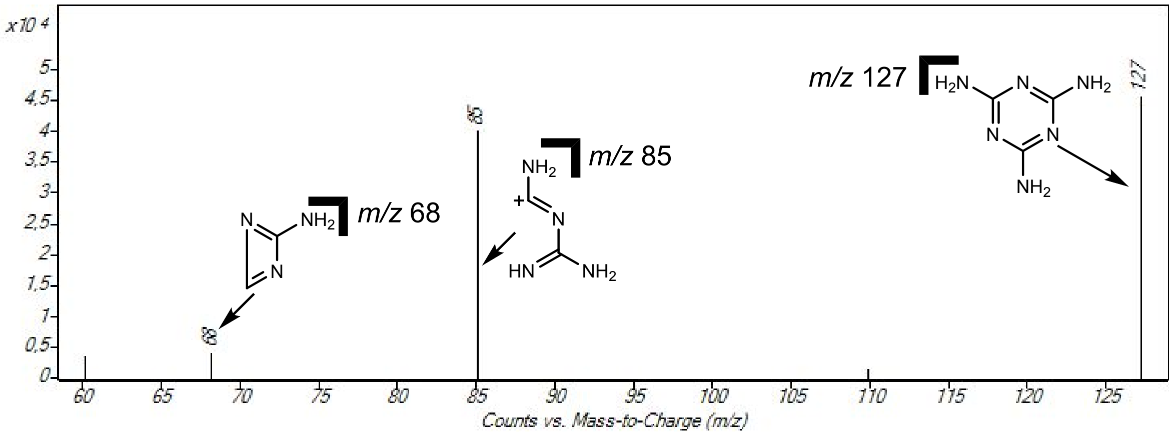

Figure S27. Product ion spectrum and proposed fragment structures of the 2-amino-4,6-dichloro-1,3,5-triazine adduct with *m/z* 127 Da formed by the reaction of 2,4,6-trichloro-1,3,5-triazine (E2) and ammonia (NH_3).

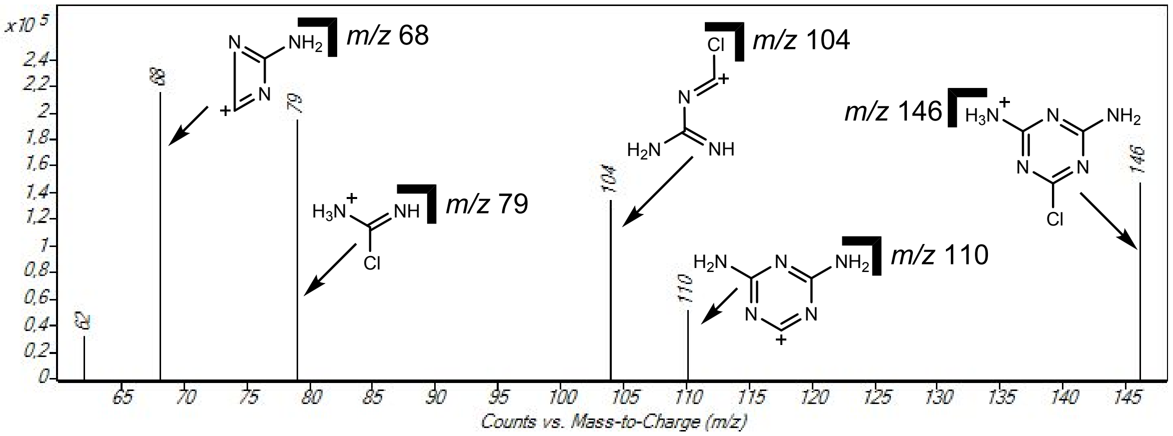

Figure S28. Product ion spectrum and proposed fragment structures of the 2,4-diamino-6-chloro-1,3,5-triazine adduct with *m/z* 146 Da formed by the reaction of 2,4,6-trichloro-1,3,5-triazine (E2) and two molecules of ammonia (NH_3).

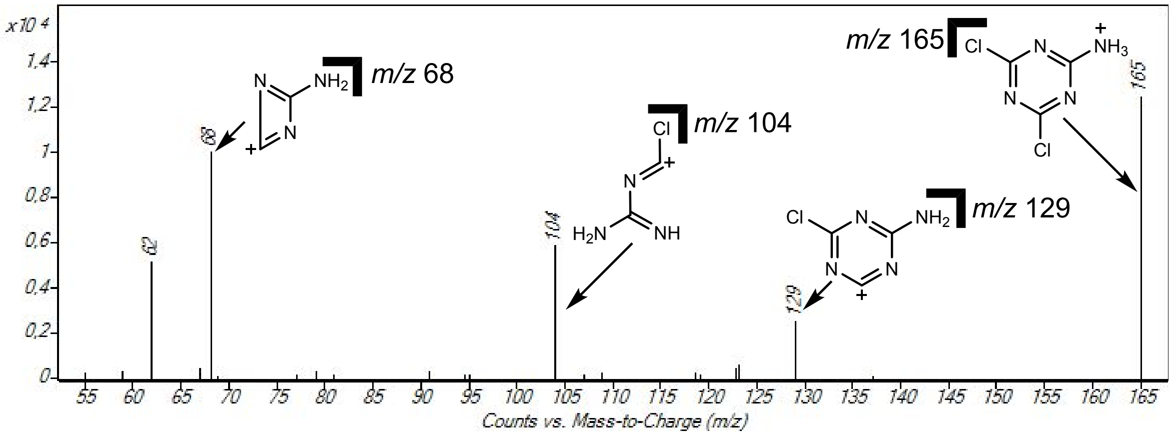

Figure S29. Product ion spectrum and proposed fragment structures of the 2,4,6-triamino-1,3,5-triazine adduct with *m/z* 165 Da formed by the reaction of 2,4,6-trichloro-1,3,5-triazine (E2) and three molecules of ammonia (NH_3).

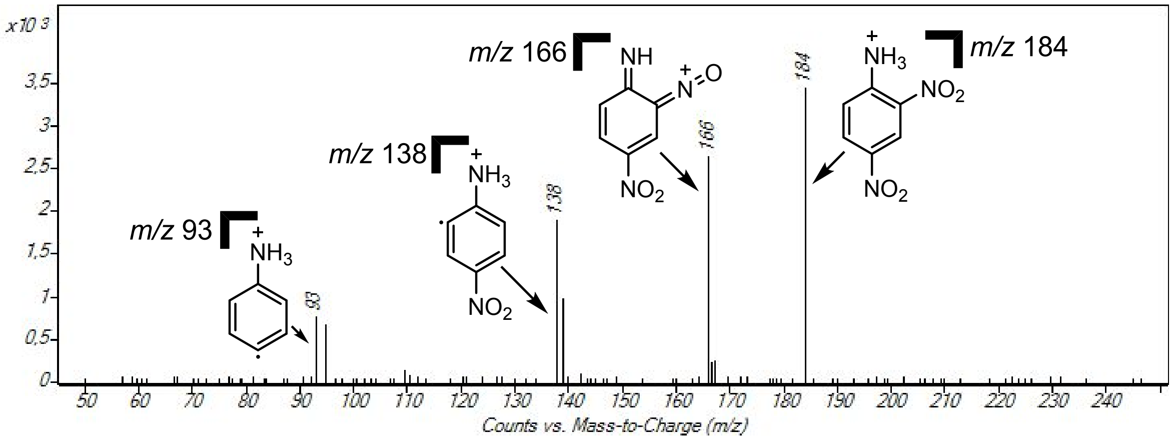

Figure S30. Product ion spectrum and proposed fragment structures of the 1-amino-2,4-dinitrobenzene adduct with *m/z* 184 Da formed by the reaction of 1-fluoro-2,4-dinitrobenzene (E3) and ammonia (NH_3).

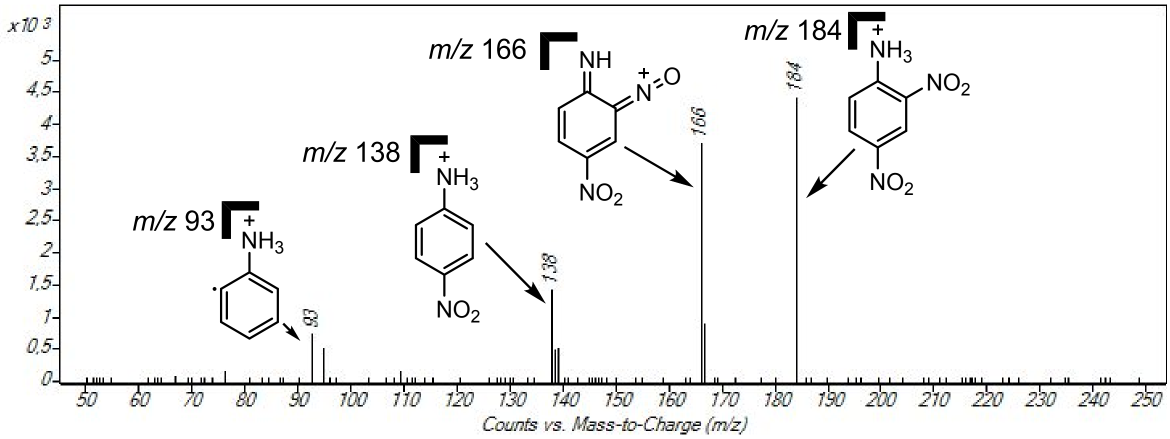

Figure S31. Product ion spectrum and proposed fragment structures of the 1-amino-2,4-dinitrobenzene adduct with *m/z* 184 Da formed by the reaction of 1-chloro-2,4-dinitrobenzene (E4) and ammonia (NH_3).

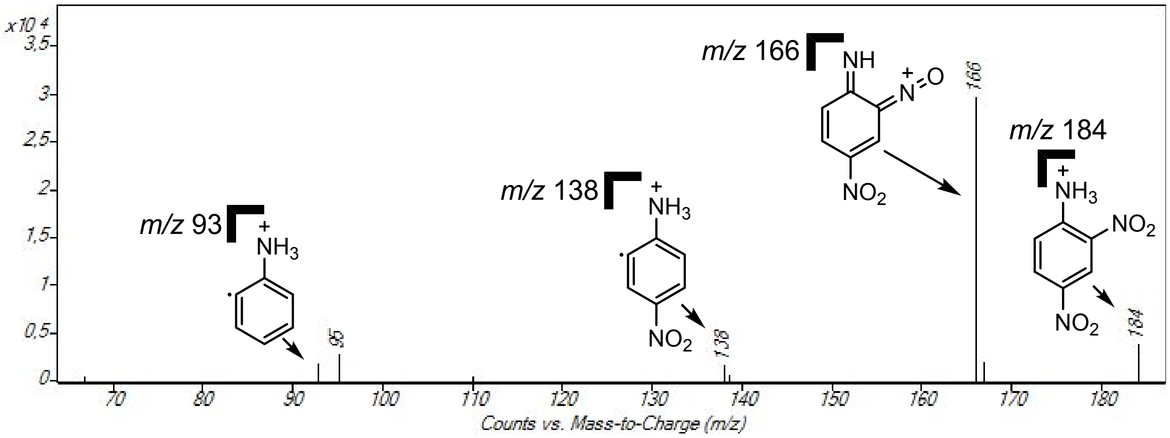

Figure S32. Product ion spectrum and proposed fragment structures of the 1-amino-2,4-dinitrobenzene adduct with *m/z* 184 Da formed by the reaction of 2,4-dinitrobenzenesulfonic acid (E5) and ammonia (NH_3).

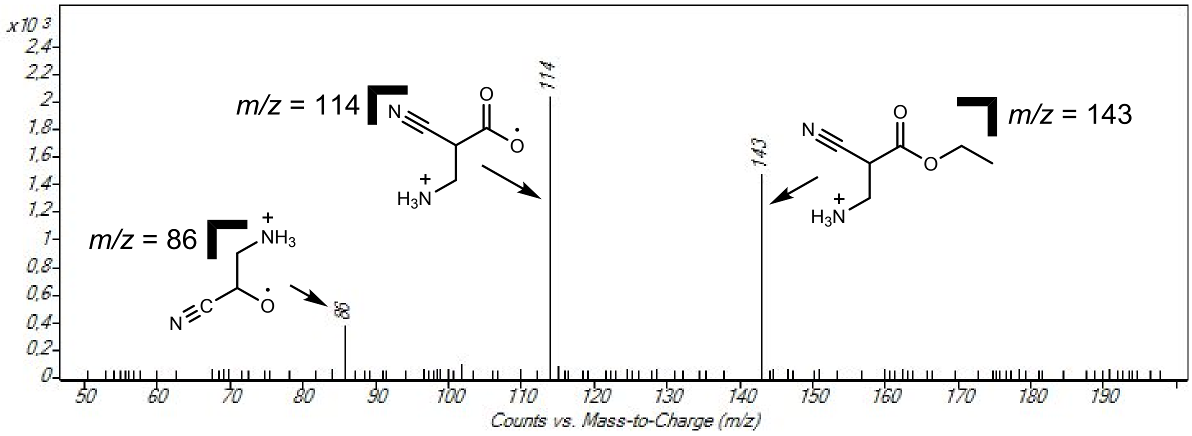

Figure S33. Product ion spectrum and proposed fragment structures of the adduct with *m/z* 143 Da formed by the reaction of ethyl 2-cyanoacrylate (F1) and ammonia (NH_3).

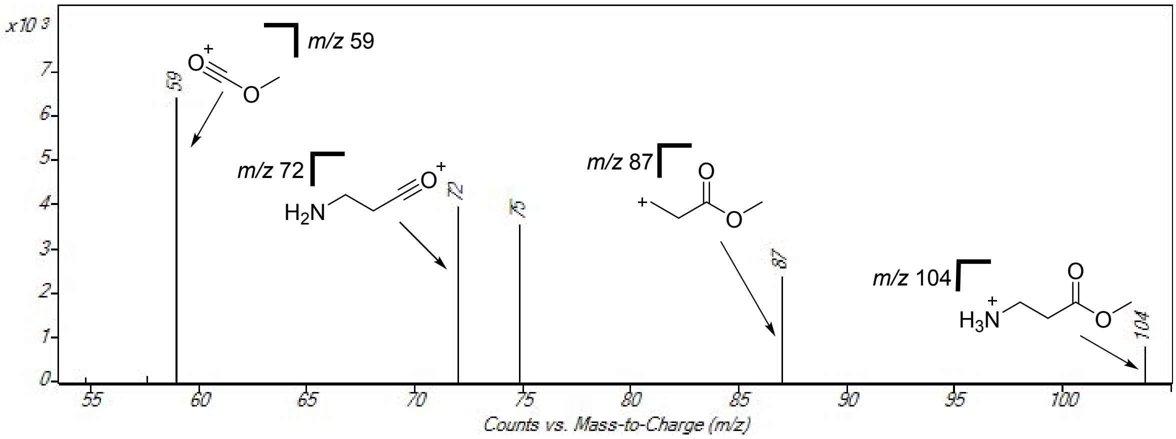

Figure S34. Product ion spectrum and proposed fragment structures of the adduct with *m/z* 104 Da formed by the reaction of methyl acrylate (F2) and ammonia (NH_3).

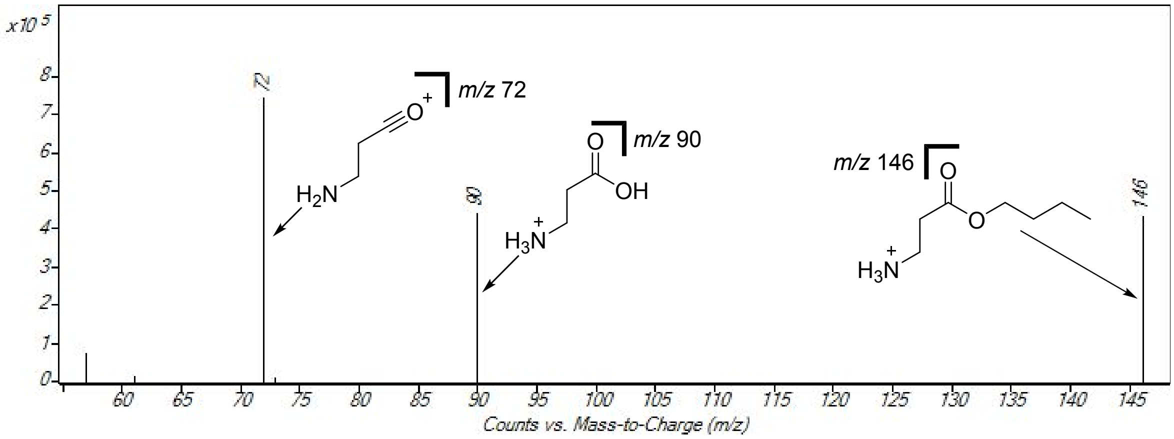

Figure S35. Product ion spectrum and proposed fragment structures of the adduct with *m/z* 146 Da formed by the reaction of butyl acrylate (F3) and ammonia (NH_3).

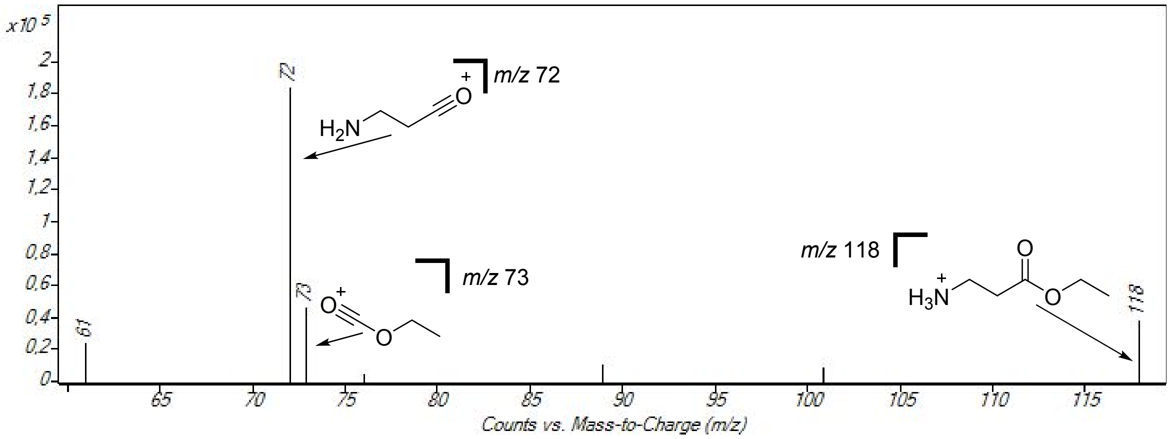

Figure S36. Product ion spectrum and proposed fragment structures of the adduct with *m/z* 118 Da formed by the reaction of ethyl acrylate (F4) and ammonia (NH_3).

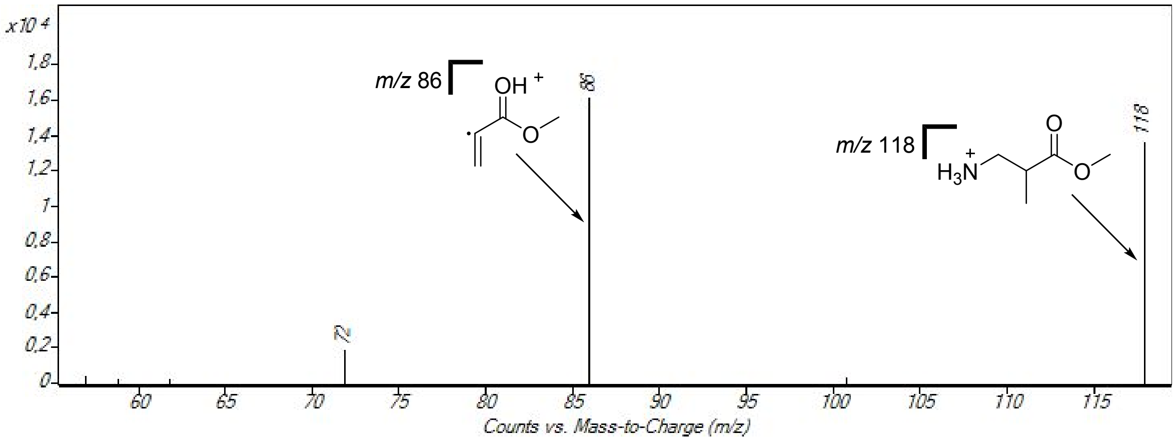

Figure S37. Product ion spectrum and proposed fragment structures of the adduct with *m/z* 118 Da formed by the reaction of methyl methacrylate (F5) and ammonia (NH_3).

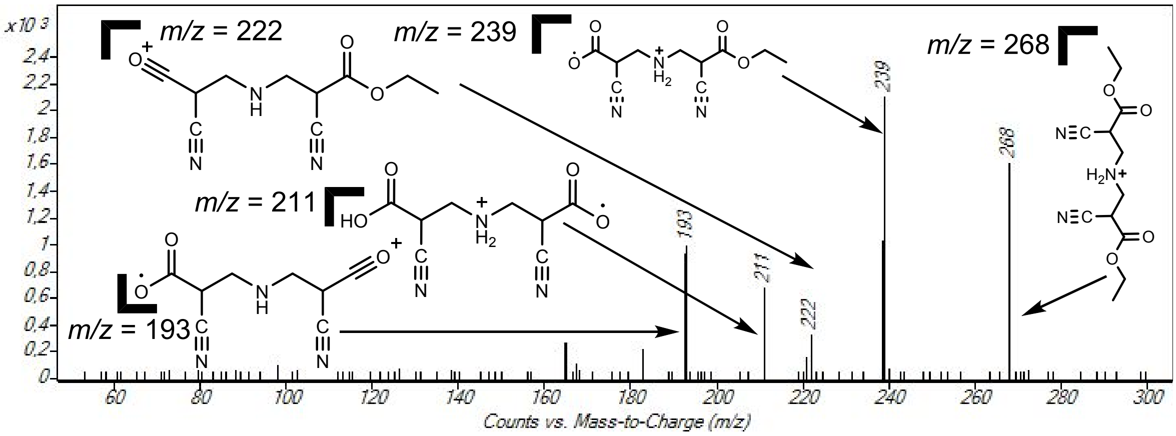

Figure S38. Product ion spectrum and proposed fragment structures of the adduct with *m/z* 268 Da formed by reaction of two molecules of ethyl 2-cyanoacrylate (F1) and ammonia (NH_3).

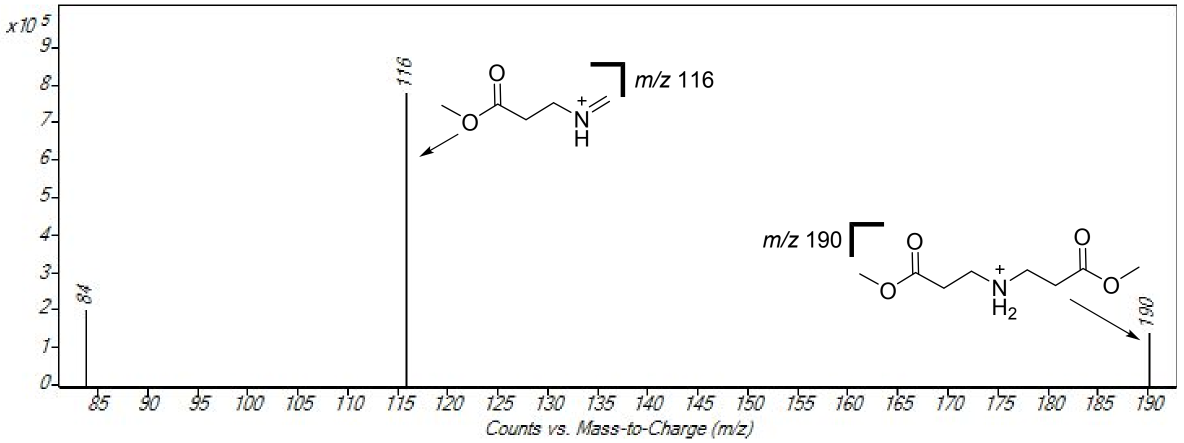

Figure S39. Product ion spectrum and proposed fragment structures of the adduct with *m/z* 190 Da formed by reaction of two molecules of methyl acrylate (F1) and ammonia (NH_3).

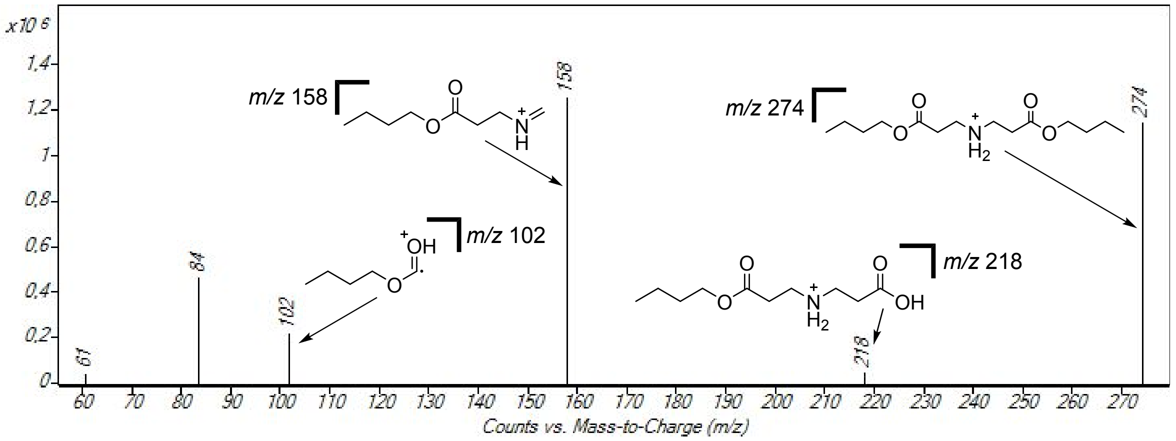

Figure S40. Product ion spectrum and proposed fragment structures of the adduct with *m/z* 274 Da formed by the reaction of two molecules of butyl acrylate (F4) and ammonia (NH_3).

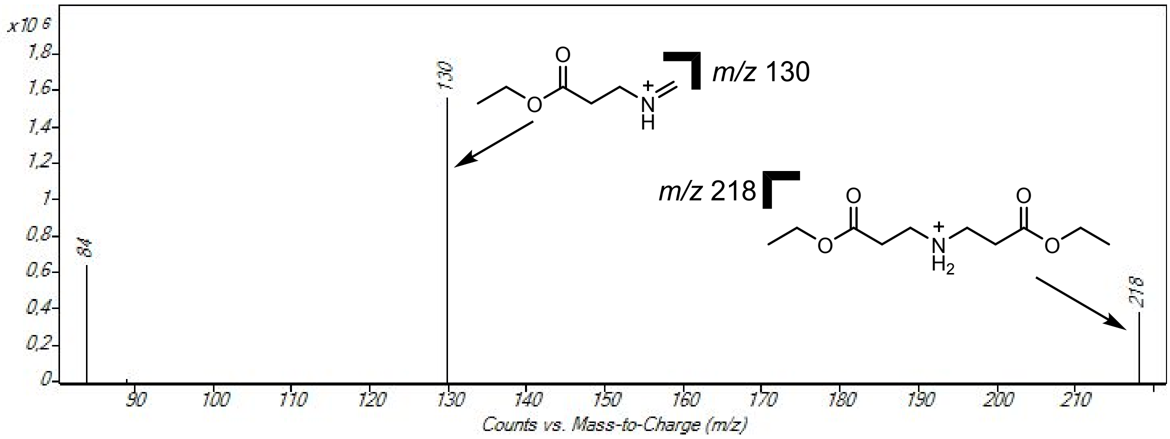

Figure S41. Product ion spectrum and proposed fragment structures of the adduct with *m/z* 218 Da formed by the reaction of two molecules of ethyl acrylate (F4) and ammonia (NH_3).

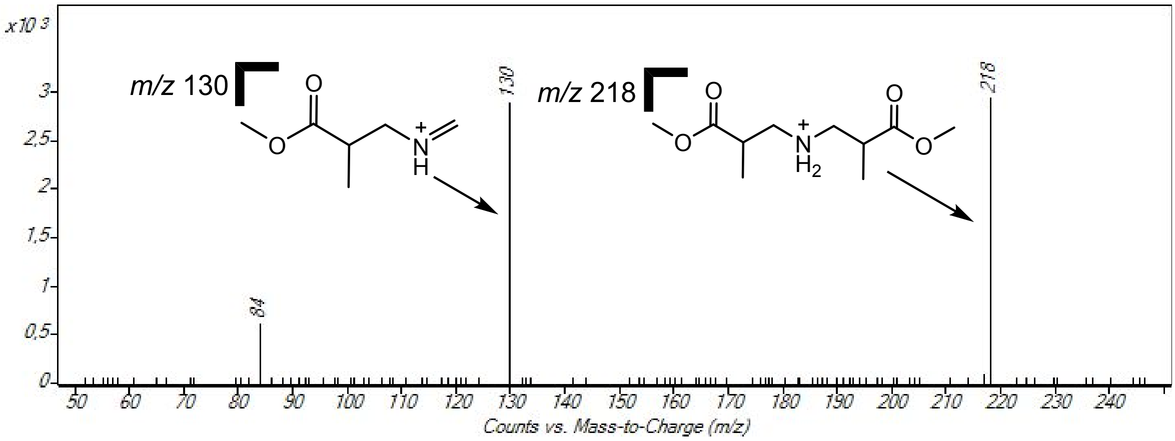

Figure S42. Product ion spectrum and proposed fragment structures of the adduct with *m/z* 218 Da formed by the reaction of two molecules of methyl methacrylate (F5) and ammonia (NH_3).

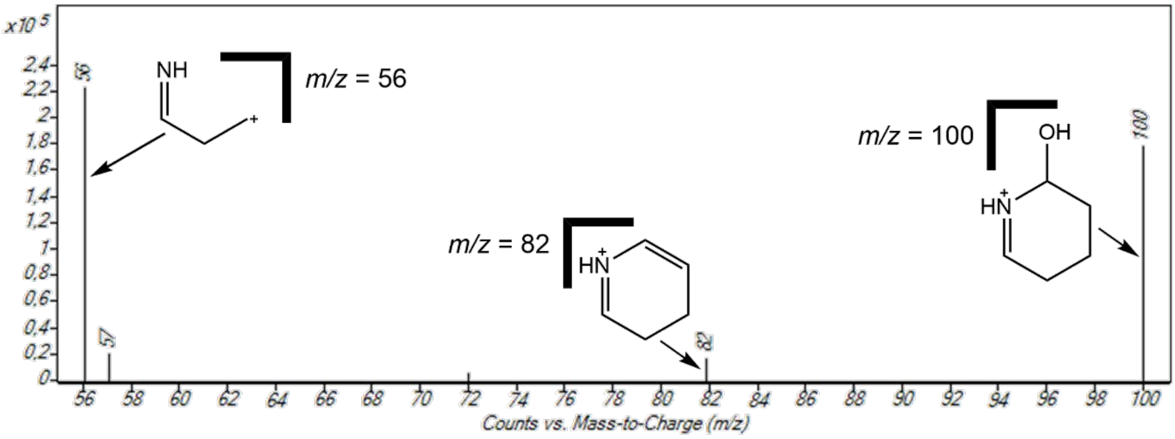

Figure S43. Product ion spectrum and proposed fragment structures of the adduct with *m/z* 100 Da formed by the reaction of glutaraldehyde (G1) and ammonia (NH_3).

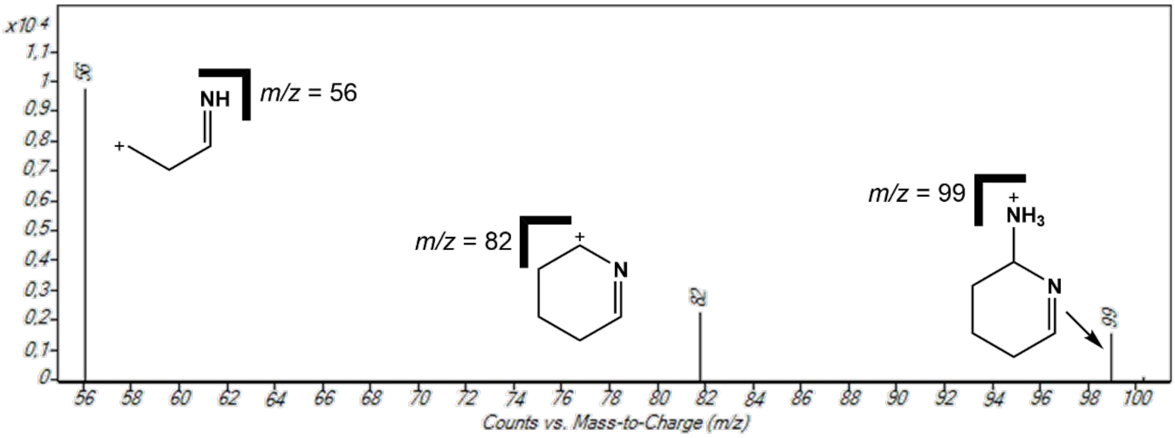

Figure S44. Product ion spectrum and proposed fragment structures of the adduct with *m/z* 99 Da formed by the reaction of glutaraldehyde (G1) and two molecules of ammonia (NH_3).

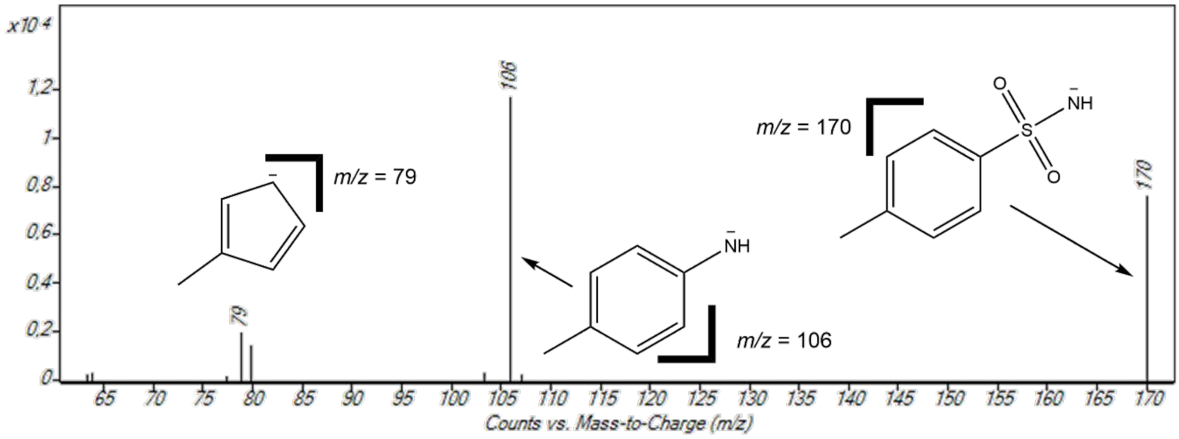

Figure S45. Product ion spectrum and proposed fragment structures of the adduct with *m/z* 170 Da formed by the reaction of chloramine T (G2) and ammonia (NH_3).

References

(1) Krutz, N. L., Kimber, I., Ryan, C. A., Kern, P. S., and Gerberick, G. F. Critical Evaluation of Low-Molecular Weight Respiratory Sensitizers and Their Protein Reactivity Potential Toward Lysine Residues. *Toxicol. Sci.* **2021**, *182*, 346–354.

(2) Sadekar, N., Boisleve, F., Dekant, W., Fryer, A. D., Gerberick, G. F., Griem, P., Hickey, C., Krutz, N. L., Lemke, O., Mignatelli, C., Panettieri, R., Pinkerton, K. E., Renskers, K. J., Sterchele, P., Switalla, S., Wolter, M., and Api, A. M. Identifying a reference list of respiratory sensitizers for the evaluation of novel approaches to study respiratory sensitization. *Crit. Rev. Toxicol.* **2021**, *51*, 792–804.

(3) Ponder, J., Rajagopal, R., Singal, M., Baker, N., Patlewicz, G., Roggen, E., Cochrane, S., and Sullivan, K. "In Litero" Screening: Retrospective Evaluation of Clinical Evidence to Establish a Reference List of Human Chemical Respiratory Sensitizers. *Front. Toxicol.* **2022**, *4*, 916370.

(4) Karol, M. H., and Kramarik, J. A. Phenyl isocyanate is a potent chemical sensitizer. *Toxicol. Lett.* **1996**, *89*, 139–146.

(5) Hopkins, J. E., Naisbitt, D. J., Kitteringham, N. R., Dearman, R. J., Kimber, I., and Park, B. K. Selective haptenation of cellular or extracellular protein by chemical allergens: association with cytokine polarization. *Chem. Res. Toxicol.* **2005**, *18*, 375–381.

(6) Pemberton, M. A., and Kimber, I. Methyl methacrylate and respiratory sensitisation: a comprehensive review. *Crit. Rev. Toxicol.* **2022**, *52*, 139–166.

(7) Pemberton, M. A., Kreuzer, K., and Kimber, I. Challenges in the classification of chemical respiratory allergens based on human data: Case studies of 2-hydroxyethylmethacrylate (HEMA) and 2-hydroxypropylmethacrylate (HPMA). *Regul. Toxicol. Pharmacol.* **2023**, *141*, 105404.
